# Obamacare: A bibliometric perspective

**DOI:** 10.3389/fpubh.2022.979064

**Published:** 2022-08-12

**Authors:** Alvaro Carrasco-Aguilar, José Javier Galán, Ramón Alberto Carrasco

**Affiliations:** ^1^Faculty of Political Sciences and Administration, Granada University, Granada, Spain; ^2^Faculty of Statistics, Complutense University, Madrid, Spain; ^3^Department of Marketing, Faculty of Statistics, Complutense University, Madrid, Spain

**Keywords:** Obamacare, Affordable Care Act, strategic diagram, PEST analysis, SciMAT

## Abstract

Obamacare is the colloquial name given to the Affordable Care Act (ACA) signed into law by President Obama in the USA, which ultimately aims to provide universal access to health care services for US citizens. The aim of this paper is to provide an overview of the political-legal, economic, social, management (or administrative), and medical (or health) repercussions of this law, using a bibliometric methodology as a basis. In addition, the main contributors to research on ACA issues have been identified in terms of authors, organizations, journals, and countries. The downward trend in scientific production on this law has been noted, and it has been concluded that a balance has not yet been reached between the coexistence of private and public health care that guarantees broad social coverage without economic or other types of barriers. The law requires political consensus to be implemented in a definitive and global manner for the whole of the United States.

## Introduction

The Affordable Care Act (ACA) signed into law by President Obama in the USA, often referred to as Obamacare, aims to provide universal access to healthcare services for US citizens. At the time of its enactment in 2010, the vast majority of the health care system was privately owned and there were 48 million uninsured non-elderly persons (1).

Previously, several initiatives had been made, including the Social Security Act of 1935, enacted by Franklin D. Roosevelt. This law established a pension system for those over 65 years of age, although in the following years it also covered the family members of workers who died prematurely, and the disabled (2). It is also worth mentioning the reform signed into law by Lyndon B. Johnson in 1965, which consisted of the creation of the two major public health services in the USA: Medicare and Medicaid. These programs were created in order to provide coverage to people with fewer resources and those who were in a state of social vulnerability. Specifically, Medicare was designed essentially for people over 65 years of age, although it also included certain vulnerable populations, which mainly covered people with limited resources (3).

From its conception to the present day, the ACA has sparked a great debate in society, obviously mainly in the USA, but also in other countries. It is undeniable that this debate is often influenced by certain interest groups that promote it, since they may have political, economic, or other interests. This study aims, as far as possible, to give an objective view of the ACA. For this reason, high-quality scientific publications will be used as a basis for the study. However, there is a large number of such publications available on this subject. In order to achieve the greatest possible objectivity given such a large amount of information, in this study we have opted to use bibliometric techniques. Although the concept of bibliometrics itself can be understood differently (4), there is a certain consensus in defining it as the use of quantitative techniques (mathematics and statistics) on the documents published on a certain subject. Thus, one advantage of bibliometric indicators is that they make it possible to measure not only the purely quantitative aspects of the subject under investigation but also its qualitative nature (5). The success of this study will be largely based on the prior documentation of the area to be investigated, since without such an understanding of the area it would be impossible to interpret the results obtained by the bibliometric analysis, which, furthermore, requires considerable refinement and a qualitative evaluation in order to obtain the definitive results.

The main objective of this study is, therefore, to provide a cross-sectional view of the implications of the ACA from its enactment in March 2010 to the present day, by comparing how it may have progressed during this period. The PEST analysis (political, economic, social, and technological) is useful for outlining the strategic environment of a region or subject matter (6). By adapting this type of analysis, this paper aims to study the political (also including related legal aspects), economic, social, management (or administrative) and medical (concerning health) aspects related to the implementation of the law, therefore, it aims to make a PESMM analysis of Obamacare. All of which in accordance with the perspective of the various high-quality scientific publications that have been published on ACA. Evidence of this type of PEST analysis using bibliographic sources is available here (6). The secondary aims of this paper fall within those typical of bibliometric analyses. As such, we also want to identify the main researchers interested in ACA in terms of countries, organizations, journals, and authors.

The rest of this paper is structured as follows. In Section Related work, a study of related research has been carried out, since other authors have conducted cross-sectional studies on the ACA, highlighting the differences between them and our proposal. Section Research methodology presents the research methodology followed. Section ACA analysis from a bibliometric perspective presents and analyzes the results of the application of this methodology in order to achieve the proposed objectives. Finally, conclusions and proposed future work are discussed.

## Related work

A search for systematic literature review (LR) or bibliometric studies (BS) related to the ACA was carried out, obtaining a current overview of these papers by restricting the search to papers published from 2020 onwards, using both Clarivate and Google Scholar. After performing the corresponding searches and studying the results obtained, the related papers have been summarized in Table 1.

**Table 1:** Related work.

**References**	**Description**	**Type**
Xu et al. (7)	Impact of ACA on colorectal cancer outcomes.	LR
Matkin and Ring (8)	Impact of the ACA on academic medical centers.	LR
Song et al. (9)	Impact of the ACA on dental health	LR
Chernew et al. (10)	The evolution of the ACA in payment systems	LR
Lee et al. (11)	Impact on women's coverage, utilization, affordability, and health after the ACA: A review of the literature.	LR
Zhao et al. (12)	Analysis of access to care across the cancer control continuum in the ACA over the past decade.	LR
Neiman et al. (13)	Impact of ACA on surgical patients.	LR
Glied et al. (14)	Study of the financial barriers of the ACA	LR
Peikes et al. (15)	Effects of the ACA on primary health care	LR
Ercia et al. (16)	Analysis of ACA in patient enrollment strategies	LR
Norris et al. (17)	Impact of ACA on the utilization of cost-sharing elimination of preventive care services.	LR
Soni et al. (18)	How ACA insurance expansion has affected health outcomes.	LR
Kamstra et al. (19)	Analysis of how the ACA fell short for a vulnerable population in Hawaii	LR
Kates et al. (20)	ACA coverage of HIV treatment and prevention funding in the USA.	LR
Nathan et al. (21)	Evaluation of the benefits of the expansion of Medicaid for oncology patients.	LR
Moss et al. (22)	Analysis of cancer care with the ACA Medicaid expansion.	LR
Hilts et al. (23)	Impact of hospital partnerships on population health.	LR
Fiedler (24)	Legislative history of the ACA	LR
Buntin and Graves (25)	Study of the evolution of health care spending since the approval of the ACA.	LR
Corlette et al. (26)	The effect of the ACA on the individual insurance market	LR
Adigun et al. (27)	Impact of the ACA on the health care of immigrants in the United States	LR
Rozier (28)	Community benefit assessment of not-for-profit hospitals in the U.S.	LR
Himmelstein and Woolhandler (29)	Analysis of medical care with the ACA	LR
Bossick et al. (30)	Impact of state legislation on reproductive health in the United States.	LR
Layton et al. (31)	Impact of ACA on long-term care.	LR
Lindley et al. (32)	Analysis of children's palliative care in the United States since 2010.	LR
Minas et al. (33)	Analysis of health disparities in oncology treatment in the ACA.	LR
Morgan et al. (34)	Study of preventing readmissions due to trauma in ICUs	LR
Marye (35)	Study of the relationship between insurance type and emergency department use for children with asthma in the United States during the ACA.	LR
Titus and Kataoka-Yahiro (36)	Analysis of barriers to health care access in Hispanics with type 2 diabetes in the ACA.	LR
Watkins et al. (37)	Analysis of the customized preventive services of the ACA	LR
Zavala et al. (38)	ACA disparities in cancer health in ethnic minorities.	LR
Hong et al. (39)	Impact of the hospital value-based purchasing program on Medicare.	LR
Clark et al. (40)	Analysis of the prevalence of health insurance among gender minorities.	LR
Steinberg et al. (41)	Racial differences in outcomes after receiving advanced heart failure therapies in the ACA	LR
Manchikanti et al. (42)	Comparative analysis before and after 2009 in patients undergoing vertebral augmentation treatment with Medicare.	LR
Ermer et al. (43)	Impact of Medicaid expansion for cancer care.	LR
Current work	Impact of the ACA at a cross-sectional level: political-legal, social, economic, medical and management.	BS

As can be seen, there are several review papers related to the ACA, but many of them are specific to certain aspects of the law, and those that are more general are not analyzed using bibliometric techniques. Based on the searches carried out, we can conclude that this paper is original and that nothing similar exists in the literature.

## Research methodology

In this section we will explain the research methodology followed in this study and the results of which will be shown in the following section.

**Figure 1:**
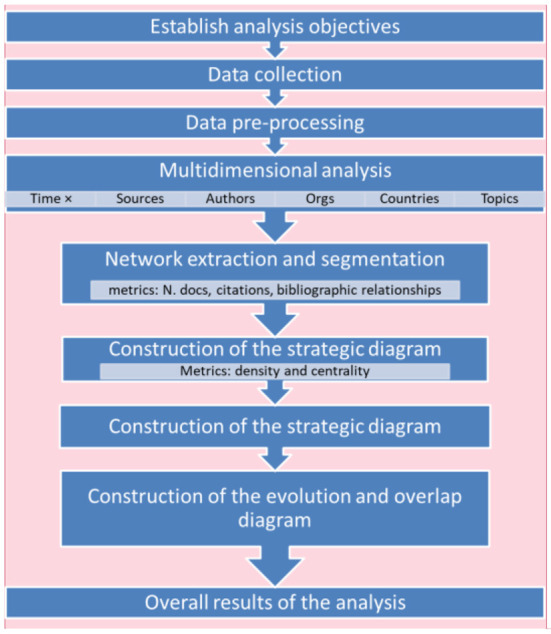
Steps used in the research methodology.

Bibliometric mapping is an important field within bibliometrics. Its objective is to show the structural and dynamic aspects of scientific research to enable further interpretation. In this paper we will follow the methodology shown in Figure 1, which is inspired by Cobo (44) and Galán et al. (45). The advantage of this methodology is that there are tools that allow us to carry out most of its stages, and in this study we mainly use the following tools: SciMAT (5), VOSviewer (46), and Microsoft Excel.

Each stage is described in more detail below.

### Setting the objectives of the analysis

This study aims to achieve the following specific objectives: to extract and analyze the different issues that have been involved in different aspects of the ACA: political, social, health, economic and management or administration, to further study the political and social aspects involved in the ACA, to study how these issues have evolved from the period of implementation of the ACA to the present-day, to identify the main researchers interested in the ACA in terms of countries, organizations, journals and authors, and to study the trend of scientific production concerning the ACA.

### Data collection

To carry out this study we required high-quality scientific literature published on the ACA. There are several bibliographic database options (45): Clarivate, Scopus, Google Scholar, etc.

In this paper, we have used Clarivate as many authors consider it to be a higher quality source, although there are fewer papers available. This is not a problem in this case, as a preliminary study has identified several thousand articles on ACA.

The time period chosen for the study will be up to 2021, not including the current year, 2022, in order for the work to be reproducible. Specifically, the chosen dates are 01/01/2008 to 12/31/2021.

In the Clarivate advanced query option, the following expression has been used on the Clarivate Web of Science Core Collection:

*TS* = *(“obamacare” or “obama-care” or “obama care” or “Affordable Care Act”)*

The TS field implies searching for publications on ACA (including different forms of spelling and informal terms) in the title, abstract and/or keywords. The query was carried out in April 2022, obtaining a total of 6,369 documents.

### Data pre-processing

The documents obtained were exported from Clarivate to seven files since only up to 1,000 documents can be exported at a time. Once this was done, they were included in SciMAT since this tool has several functionalities for data preprocessing. To increase the quality of the data, the keywords have been normalized by merging them in their plural and singular forms, words have also been merged with their corresponding synonyms, and several keywords that refer to the same concept have been identified by using the Levenshtein distance in SciMAT.

### Multidimensional analysis

To obtain an overall view of the subject under analysis, we are going to perform the analysis from different points of view or dimensions. Table 2 explains each of these dimensions and includes the types of quantitative analysis we are going to perform on them, which are typical of the tools we are using in this paper (SciMAT and VOSviewer).

**Table 2:** Dimensions of analysis, meaning, and type of analysis available for them.

**Dimension**	**Description**	**Type of analysis**
Sources	Journals, conference proceedings…	Citations
Authors	Author and co-authors	Co-authorships
Organizations	Authors' affiliations and funding organizations	Co-authorships
Countries	Authors' countries	Co-authorships
Topics	Keywords	Co-occurrences

According to the type of analysis, we use the following bibliographic relationships that will allow us to quantitatively relate various bibliographic elements, thus allowing us to construct the corresponding networks (45); co-authorship, the relationship of the articles is determined according to the number of co-authorships; citation, the relationship of the articles is determined according to the number of times they cite each other; co-occurrence, the relationship of keywords is determined according to the number of documents in which they appear together. For this purpose, the equivalence index is often used.

### Network extraction and segmentation

**Figure 2:**
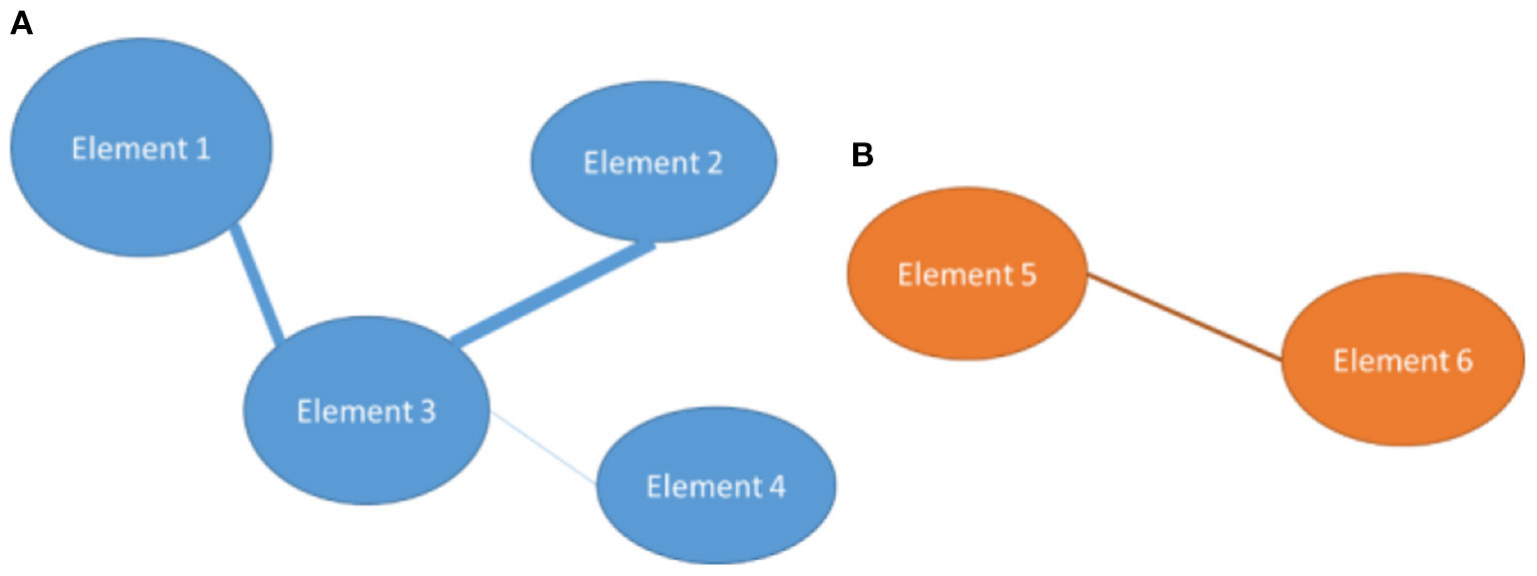
Thematic network where the different elements (dimensions) and segmentation of the network are linked using a different color for groups, group **(A)** and group **(B)**.

After this construction, a process of segmentation or grouping of the elements that are considered similar is usually performed. Therefore, nodes that are considered to be close enough to each other and sufficiently separated from the other groups are grouped together. An example of a thematic network can be seen in Figure 2.

### Construction of the strategic diagram

This type of diagram is particularly interesting in the analysis of co-occurrences as it allows the importance of each of the topics that have emerged in the analysis to be outlined. It is based on two measures (5):

- **Centrality** measures the degree of interaction of a network with other networks. This value can be understood as a measure of the importance of an element in the development of the entire analyzed field of research.
- **Density** is the internal strength of the network or element in question. This value can be considered to be a measure of the degree of the development of the topic.

**Figure 3:**
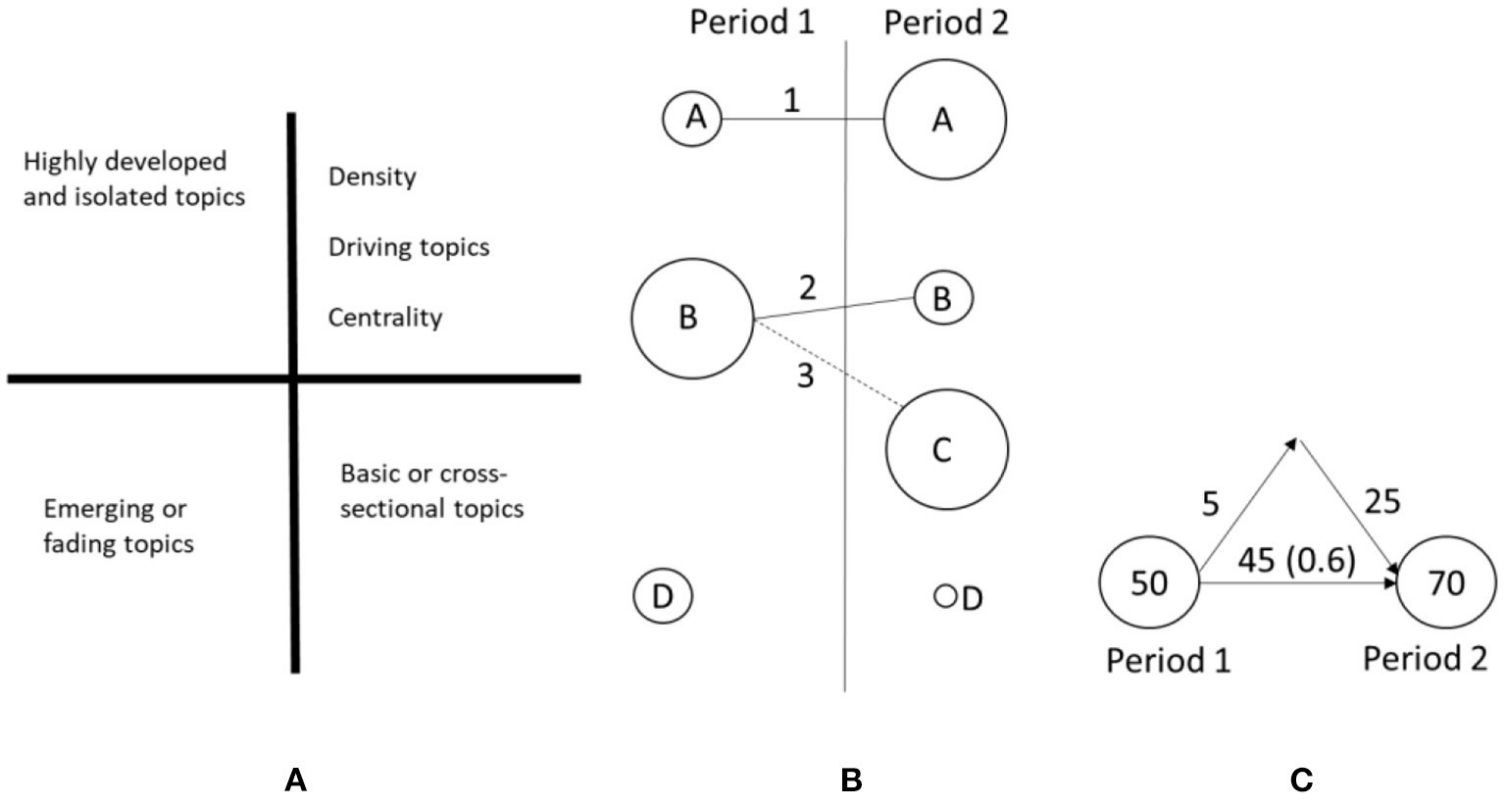
Strategic diagram **(A)**, examples of an evolution diagram **(B)**, and overlap diagram **(C)**.

The strategic diagram makes it possible to classify the topics that were identified in the bibliometric study into four categories, as shown in Figure 3.

### Construction of the evolution and overlap diagram

The topic divisions have been divided by periods, and afterwards, the evolution and overlap diagram of the corresponding items is obtained, which shows the evolution of the topics and the overlapping of these detected topics (which is an indicator of their stability) in successive periods of time. An example of both diagrams is shown below in Figure 3.

### Visualization and interpretation of results

Based on the results obtained for each dimension, mainly the graphs already mentioned in the previous points, the researcher must interpret and, in many cases, fine-tune the results based on the selection of the various parameters that the tools have, until they understand what is happening in that particular analysis.

### Overall results of the analysis

Although this methodology is largely based on quantitative techniques, the researcher, based on the different global results obtained for each dimension (subject, authors, countries, etc.), has to interpret them globally and try to meet the objectives set out in the first stage.

## ACA analysis from a bibliometric perspective

In this section we will explain the results of applying the previously mentioned research methodology to the topic analysis. To do so, we will begin by analyzing the different topics related to the ACA that were identified in the bibliometric analysis and we will try to look at them from the different PESMM perspectives mentioned above. In this analysis, an analysis of the temporal evolution of these issues must be carried out, since the application of the law has evolved with the different changes of government, judicial resolutions, etc. Subsequently, we will analyze the different sources (journals, conference proceedings, etc.) where we have found studies related to the ACA. We will then analyze the contributions made to this area by the most important authors, countries and organizations. Finally, we will extract the overall results of the analysis and extract the fundamental factors of the intended PESMM analysis.

### Topic analysis

As mentioned above, this type of analysis is essential in order to meet the main objectives of this study. As such, we will analyze the keywords of the articles obtained in three different ways: those specified by the authors themselves; those specified by the Clarivate for each article; and the additional keywords extracted from the title of the article and the abstract. It is important to remember that for this type of analysis we will use the co-occurrences of these keywords among the different articles, as mentioned in Section Research methodology. In other words, one keyword will be related to another according to the number of documents in which both appear together.

We will divide the period of analysis in two, since the ACA itself has undergone several variations from its conception to its attempted implementation, motivated among other factors by the complexity of the implementation itself, judicial vicissitudes… but above all, one such determining factor has been the different changes in the U.S. governments:

- **Until 2017**: would be the stage of growth in scientific interest in this subject, including work prior to the law itself and the implementation period under the Obama administration. Obviously, this administration has been a great promoter of this law, so its development during this period is quite noticeable.
- **2017–2022**: is the post-Obama stage, which included that of President Trump and later Biden. This stage was also influenced by the pandemic caused by Covid-19. The political changes after the 2016 elections brought about reforms to the law that generated uncertainty, although after Biden's arrival to power the law now seems to be more entrenched.

For the analyses shown below, the SciMAT tool will be used primarily for the construction of the strategic and evolution/overlap diagrams (explained in Section Research methodology). Note that prior to the construction of these diagrams, the keywords have been preprocessed by grouping synonymous terms (although written differently) as explained in Section Data pre-processing.

#### Period of analysis “until 2017”

**Figure 4:**
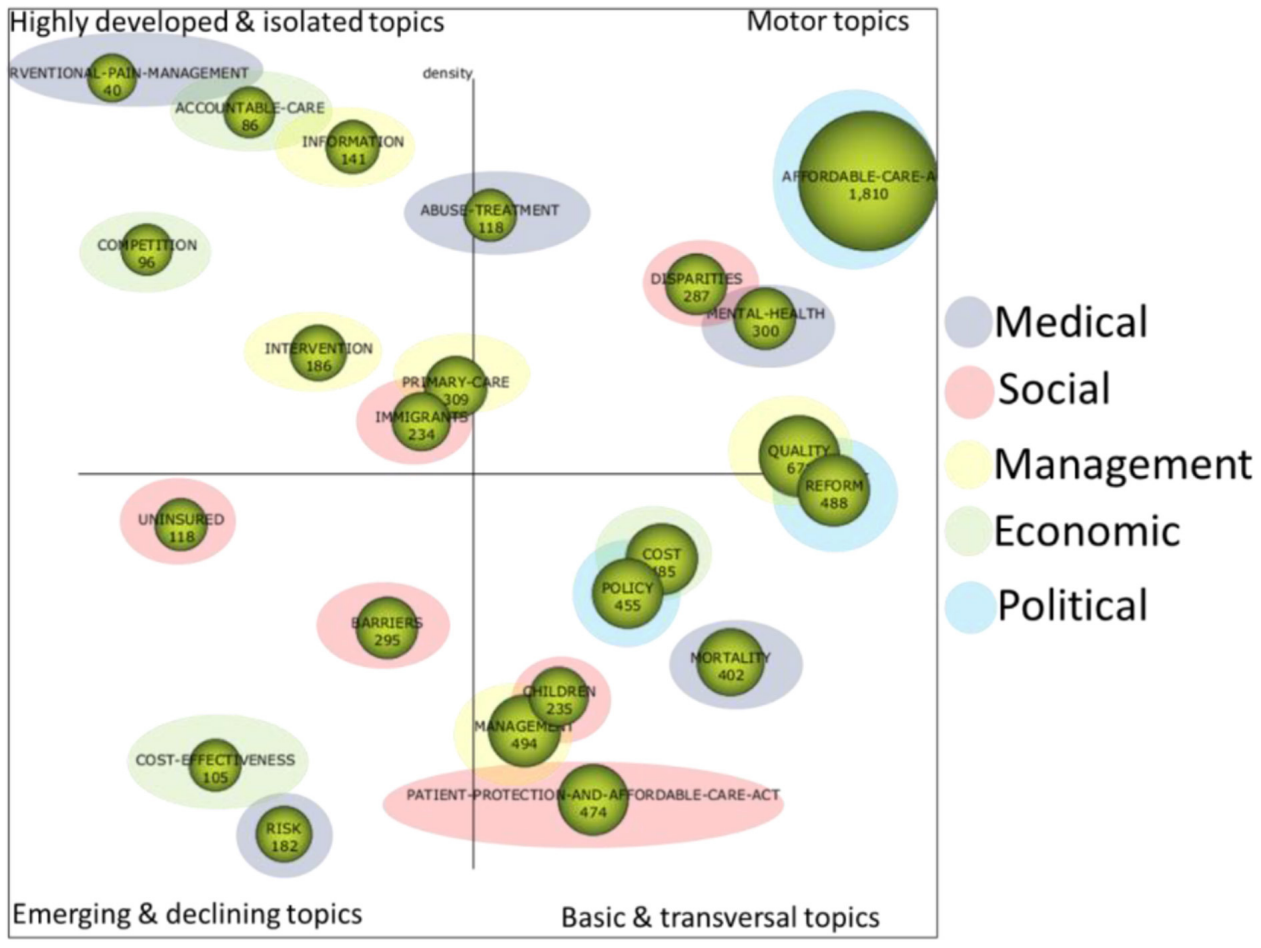
Strategic diagram analysis period “until 2017”.

Figure 4 shows the strategic diagram for the first analysis period “until 2017.” This diagram was initially constructed with SciMAT and was enhanced with the typology of each quadrant in order to determine the importance of the topics. In addition, each of the topics has been qualitatively typified into five major areas or topic categories, each of which is related to the ACA:

- POLITICAL: this includes topics dominated by the political and legal aspects of the health law reform implemented by the ACA.
- ECONOMIC: issues related to the economic aspects of the ACA are included in this category.
- SOCIAL: the social impact of the ACA is unquestionable and this category includes issues related mainly to the social aspect of the law.
- MANAGEMENT: this includes issues related to the management and administration involved in the implementation of such a complex law that has such a significant impact.
- MEDICAL: the health of citizens was of course the primary objective of the law and studies with medical implications of the law would fall into this category.

##### Political

In this category we have included the following topics:

- *AFFORDABLE-CARE-ACT* (Supplementary Figure 10). This is the most important (it is a driving topic) and extensive thematic network of the period, bringing together works of various kinds related to the ACA law itself. A significant work included here is President Obama's own analysis in an initial paper on the Obama-Biden reform (47) and a later paper on the implementation of the reform and the steps to be taken next (48). Studies on MEDICAID are also included, which is a government-run health coverage offered to people with limited income. The extension or expansion of this type of coverage is one of the main objectives of the ACA, and thus we can find works that deal with this expansion in low-income adults (49).
- *REFORM* (Supplementary Figure 11). This is a basic and cross-sectional issue that essentially includes political and legal aspects related to the law reform, which has involved important studies (50–53). The changes in insurance coverage brought about by the law would also be included here, with a number of studies, such as (54). There are also interesting studies that consider whether the law reform will lead to structural or circumstantial changes, such as the one in (55), which is closely related to the hypothesis set out in this paper.
- *POLICY* (Supplementary Figure 12). This is also a basic and cross-sectional topic that essentially includes aspects of studies that are more closely related to politics in this period (56–59). Studies on public opinion are also included in this topic (60).

##### Social

In this category we have included the topics explained in more detail below:

- *IMMIGRANTS* (Supplementary Figure 13). Undoubtedly, this is one of the groups that is most affected by the ACA. We can find studies on undocumented immigrants (61), Hispanics (62), the abolition of immigration-related barriers to health care (63), etc.
- *DISPARITIES* (Supplementary Figure 14). In this topic we can find papers related to disparities based on ethnicity, particularly in the case of African Americans (64), gender (22), rural areas (65), etc.
- *UNINSURED* (Supplementary Figure 15). The uninsured are a serious problem addressed by the ACA. As examples we have the following studies (66, 67).
- *BARRIERS* (Supplementary Figure 16). Studies would include other types of barriers to health access such as language, educational level, teenagers' access to contraceptives (68), etc.
- *CHILDREN* (Supplementary Figure 17). Here we find interesting studies on health management in children (69) and school cooperation (70).
- *PATIENT-PROTECTION-AND-AFFORDABLE-CARE-ACT* (Supplementary Figure 18). This last social issue deals with various topics related to the protection of patients' health, such as a healthy diet (71), access to accurate health information on the Internet (72), tele-assistance (73), etc.

##### Economics

In this category we have classified the following topics:

- *COMPETITION*. An interesting topic to analyze in terms of the ACA is the impact that the law has had on the health care market (74), antitrust policies (75), etc.
- *ACCOUNTABLE-CARE*. This topic is closely related to the issues of equipping staff in organizations made possible by the law (76).
- *COST*-*EFFECTIVENESS*. Addresses economic efficiency in the health care industry (74).
- *COST*. Similar to the previous topic, it focuses on the changes in the costs implied by the law, such as the elimination of certain shared costs (77).

##### Management

In this category we have classified the following topics:

- *QUALITY*. The issue of quality associated with the health care system is a critical topic that has been addressed, for example, by (78).
- *PRIMARY-CARE*. Primary health care is key for health care management, in this regard we can find several studies, such as (79).
- *INTERVENTION*. An important aspect of management is the reduction of readmissions (80), treating the mental health of caregivers (81), etc.
- *INFORMATION*. Communication is a key part of health management, with regard to both the ACA itself and the health issues included in it, such as proper nutrition, in which we highlight studies related to fast food (82), medication (83), etc.
- *MANAGEMENT*. This topic covers the management process itself in relation to various aspects of the ACA, highlighting the work of (84) on the transition to reform brought about by the ACA.

##### Medical

In this category we have included all topics related to ACA and various medical matters:

- *INTERVENTIONAL-PAIN-MANAGEMENT*. This topic deals with pain management and pain treatment processes including treatments for chronically ill patients, e.g., pain management and pain management processes (85).
- *MORTALITY*. These are studies that deal with mortality related to diseases such as cancer (86), cardiovascular diseases (87), etc.
- *RISK*. Health risk management is an important topic that includes preventive treatment of patients (88).
- *ABUSE-TREATEMENT*. Drug abuse is a challenge for the ACA with important work such as (89).
- *MENTAL-HEALH*. Psychiatric disorders, behavioral disorders, depression, etc. are included here with various studies such as (90), which deals with access to this type of care according to sociodemographic profiles. An interesting study is (91), which deals specifically with the relevance of ACA in improving citizens' mental health.

#### Analysis period “2018–2021”

**Figure 5:**
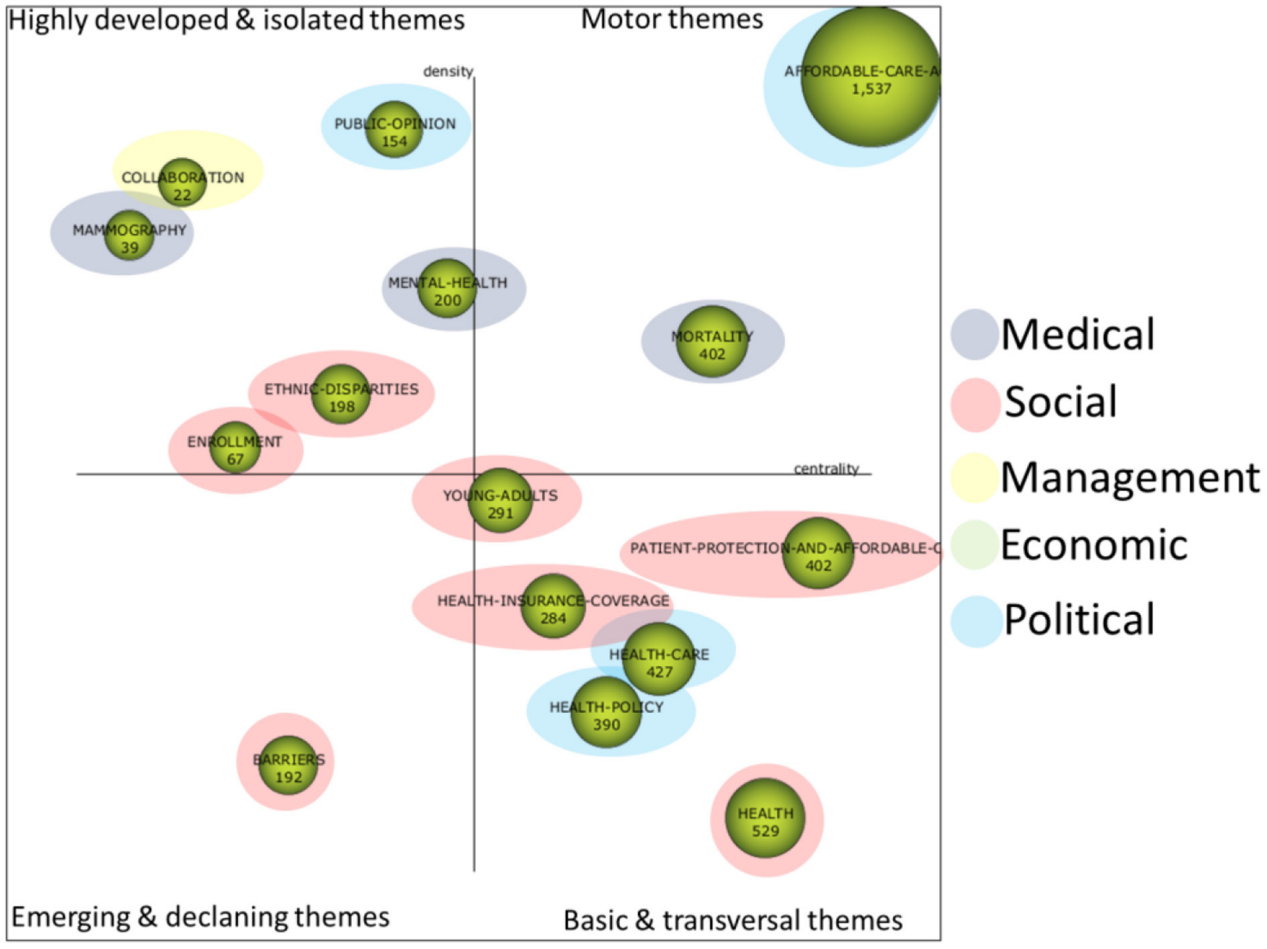
Strategic diagram analysis period “2018–2021”.

The extended strategic diagram for this period can be found in Figure 5. We can make the same observations about its construction as we have made for the previous period (see Figure 4). It should be noted that in this period we have only been able to classify one thematic group in the area of economics. It is not that there are no studies that include economic aspects related to the ACA, but rather that they are not connected closely enough for the analysis to group them together. Therefore, certain economic aspects are less prevalent in the other areas in the current period.

Each of these categories is explained in more detail below.

##### Politics

In this category we have included the topics explained in more detail below:

- *AFFORDABLE-CARE-ACT* (Supplementary Figure 19). This thematic group has the same meaning as the one studied in the previous period and, similarly to the previous period, in the current period it is also the most important in terms of the number of documents and their importance, qualifying as a driving topic in the strategic diagram. Debate continues over what the near-term future of the reform (92) and the expansion of health coverage will be since the arrival of the Biden administration (93).
- *HEALTH-POLICY* (Supplementary Figure 20). This is a basic, cross-sectional topic that addresses issues such as better health brought about by the reform (94), community needs with respect to hospitals (95), equality in terms of health (96), etc.
- *HEALTH-CARE* (Supplementary Figure 21). This network includes issues such as Americans' own understanding of the welfare state (97) and the specific challenges that lie ahead for the welfare reform.
- *PUBLIC-OPINION* (Supplementary Figure 22). The law itself still has an uncertain future, and in this regard public opinion has become particularly important during this period, as the public's view will undoubtedly dictate the future of what will happen with the ACA. We found several studies in this regard (98), some of them conducted in the era of Donald Trump (99).

##### Social

The following is an explanation of the topics included in this category during this period:

- *ETHNIC-DISPARITIES* (Supplementary Figure 23). This includes studies related to the disparity in the health care system in relation to migrants (100), Hispanics (101), etc.
- *YOUNG-ADULTS* (Supplementary Figure 24). There are several studies that deal specifically with young people and adults (102, 103).
- *HEALTH-INSURANCE-COVERAGE* (Supplementary Figure 25). As was seen in the first period of analysis, the impact of the ACA on insurance coverage is an important topic of analysis. In the current period, we have found studies focused on retirees (104, 105), etc.
- *BARRIERS* (Supplementary Figure 26). Again, as in the previous period, this group of topics is focused on the study of barriers to the expansion of medical care (106), disparities in access for the disabled (107), etc.
- *PATIENT-PROTECTION-AND-AFFORDABLE-CARE-ACT* (Supplementary Figure 27). This group of topics is also found in the previous period. It includes papers on the health of the uninsured (108), financing treatment of cancer patients (109), effects of the law on the management of chronically ill patients (11), etc.
- *HEALTH* (Supplementary Figure 28). This topic includes analyses of people with limited income (110), and the treatment of those addicted to certain substances (111), etc.

##### Economics

In this category we have classified the following topics:

- *ENROLLMENT* (Supplementary Figure 29). In this period, which includes the Trump era, there are several studies that deal with the issues related to the recruitment of policyholders in the ACA plan (112, 113).

##### Management

In this category we include a single topic:

- *COLLABORATION*. It includes collaborations between organizations to manage health (114, 115), including not-for-profit hospitals (28).

##### Medical

It includes various medical topics related to the ACA:

- *MORTALITY*. The subject of mortality was also included in the previous period; some papers on this subject can be found in (116). It also includes studies related to Covid-19 (117).
- *MENTAL*-*HEALTH*. This is another topic that was also found in the previous period. Some studies that could be cited in this regard are (118, 119).
- *MAMMOGRAPHY*. In this period this medical test has become very important due to its global advancement in the health system as a preventive method, some working examples are (120, 121).

#### Topic development from the period “until 2017” to “2018–2021”

**Figure 6:**
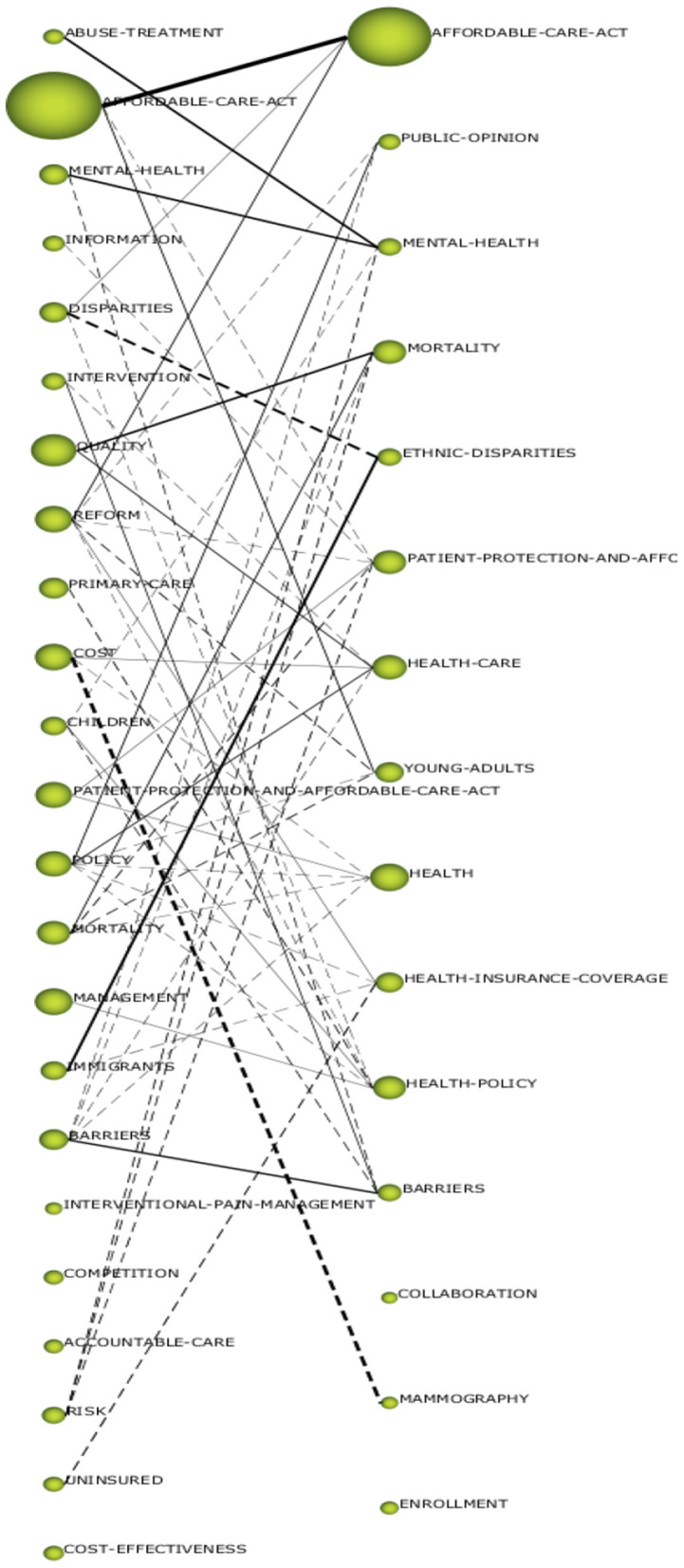
Evolution diagram from the period “Until 2017” to the period “2018–2021”.

The following evolution diagrams have been made in order to study this development (Figure 6). This diagram shows the topics that have remained relevant from one period to the next (*AFFORDABLE-CARE-ACT, BARRIERS, DISPARITIES, MORTALITY*, and *MENTAL-HEALTH…*) and which have already been discussed in the previous points. Several of the topics from the first period have been dropped, especially those related to the economic aspects of the ACA, such as *COMPETITION, COST, COST-EFECTIVENESS*, etc.

In the last period, some new topics have appeared, such as *MAMOGRAPHY, ENROLLMENT, COLLABORATION* which may imply a specialization of studies in a law that has become more widely implemented. In the overlap diagram (see Supplementary Figure 30) we can quantitatively see how 21% of the topics have remained the same from one period to the next and how the second period showed a drop in the number of topics covered.

### Source analysis

**Figure 7:**
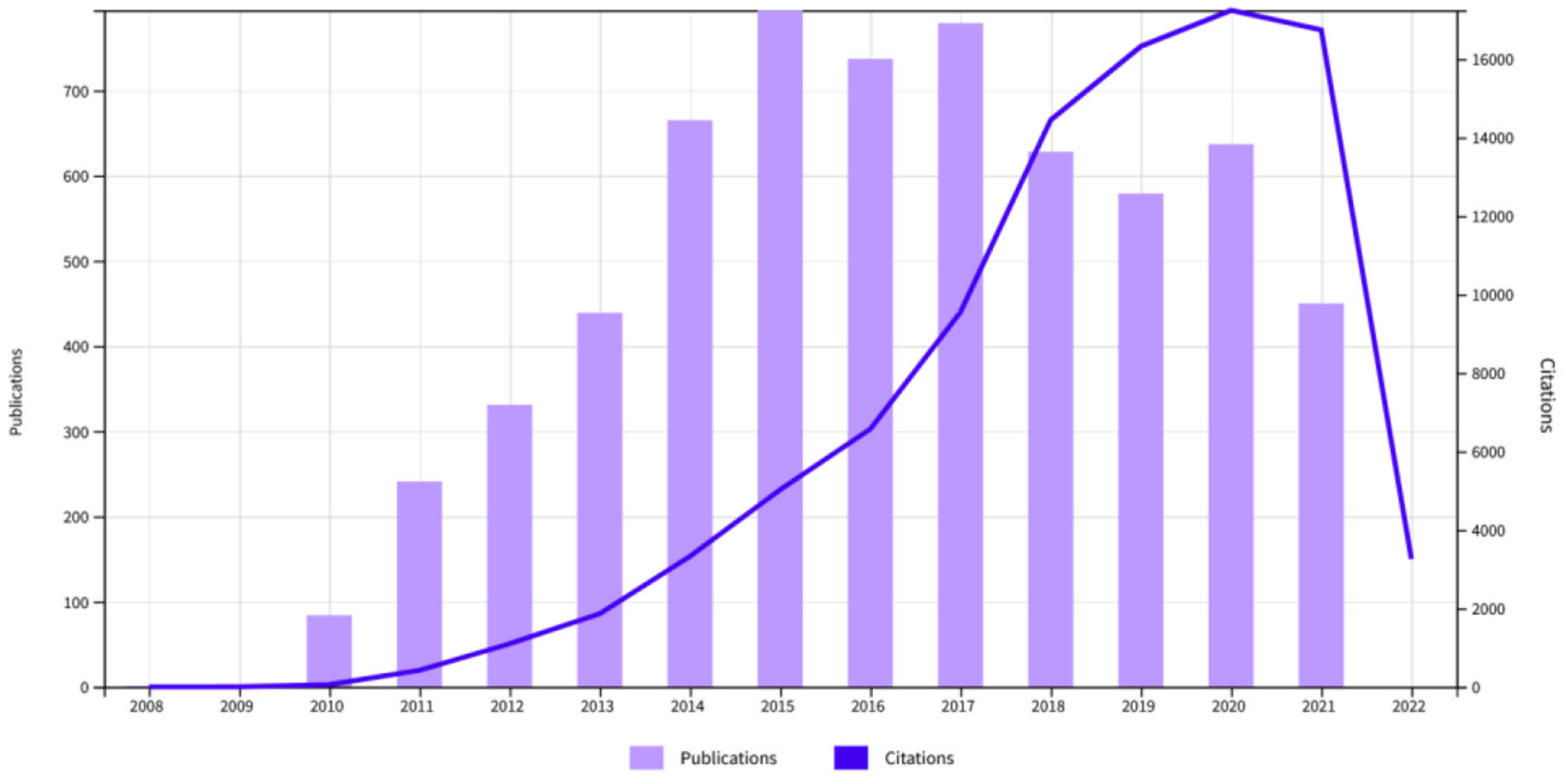
Changes in the number of publications and citations on the subject.

We will first study how publications on the subject have evolved over time. Figure 7 shows this evolution both in terms of publications and citations. An initial conclusion is that the topic has been of growing interest in the scientific community up until 2017, which was the last year of the Obama administration. This fact is evidenced by the number of publications. If we look at the quotations, we can also see a shift in the cycle that occurs in that same year, where the quotations stop growing exponentially and finally end up decreasing from 2020 onwards. As this change in trend occurred in 2017, it is ruled out that the decline in publications and general interest in ACA is due to the effects of the pandemic caused by COVID-19.

If we want to get an idea of the type of studies being carried out on the subject, it is interesting to look at the areas in which the different publications have been produced. We can observe these areas in (Supplementary Figure 31). As we can see, the areas related to health policies are the most predominant, but areas such as medicine, economics, society and law are also important.

Supplementary Figure 32 shows the typology of the publications analyzed, in which scientific articles clearly stand out, followed by editorials and conference abstracts. It is also worth mentioning that 35 review books related to the subject have been found.

### Author analysis

The most important authors in terms of the number of publications are shown in (Supplementary Figure 33). As can be seen, Professor Ben Sommers of Harvard University and a specialist in Economic Policy and Health is by far the most named with 87 publications related to the ACA. He is a major author with a 54-h-index (i.e., he has 54 publications with at least 54 citations) and with more than 11,000 citations at the time of this paper, according to Google Scholar, and whose main research interests are health policies for vulnerable populations, the uninsured, and the social security system. He has received numerous awards for his research, including *Outstanding Dissertation Award*, the *Alice Hersh New Investigator Award*, the 2015 *Article-of-the-Year Award* and the 2017 *Health Services Research Impact Award* from *Academy Health*, a preeminent national association of health policy researchers, according to its website.

Figure 7 shows the relationship of publications and the link between authors determined by the number of papers they co-signed.

### Country analysis

**Figure 8:**
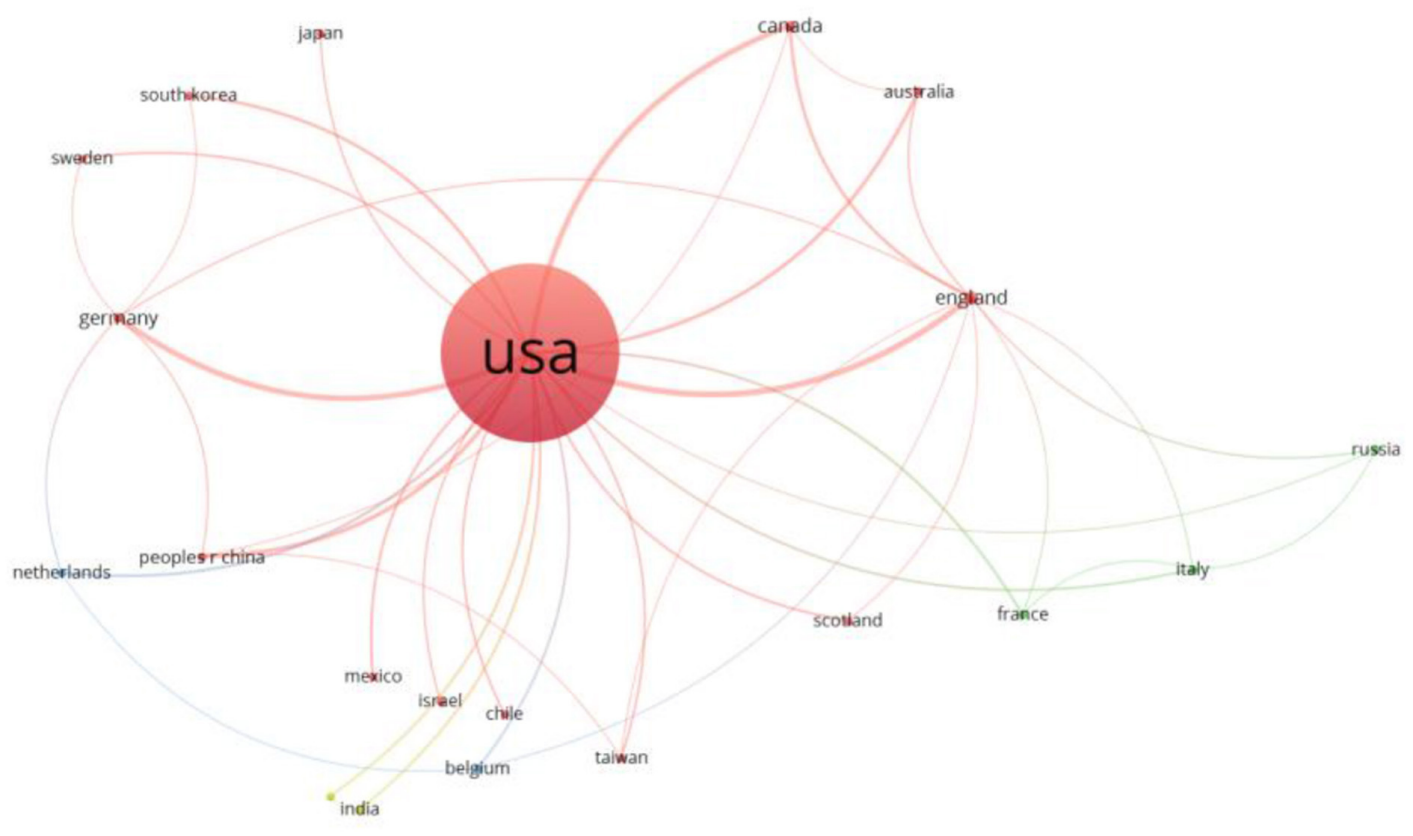
Co-authorship network of the most important countries in the subject analyzed.

The country analysis shows that the USA has taken the lead in this area, as can be seen in Figure 8. The ACA has also sparked some interest in the United Kingdom, Canada, Germany, and China.

Likewise, we can build a network of co-authorships according to countries, as we did previously for the authors. This network is shown in (Supplementary Figure 35). Countries with the highest number of publications on the analyzed topic, which again corroborates the importance of the United States in this type of study, which is only natural since it is a law that is specific to this country.

### Analysis of organizations

As this is a topic of great interest in the USA, there is an extremely high number of public and private universities and research centers in the USA that have publications on this subject. Among the most important organizations in ACA research are Harvard University, the University of California system, which is composed of several universities in California, the University of Michigan, and the University of Pennsylvania, as shown in Figure 8.

If we build a network of co-authorships between organizations to give us an idea of the collaboration between them, we will observe that there are many instances of collaboration between the different institutions in the USA when it comes to analyzing the ACA. This network is shown in Supplementary Figure 37. Organization analysis by author.

We can also conclude from this network that organizations belonging to the same system are obviously more likely to collaborate with each other in a more active way.

### Overall results of the PESMM analysis of the ACA

**Figure 9:**
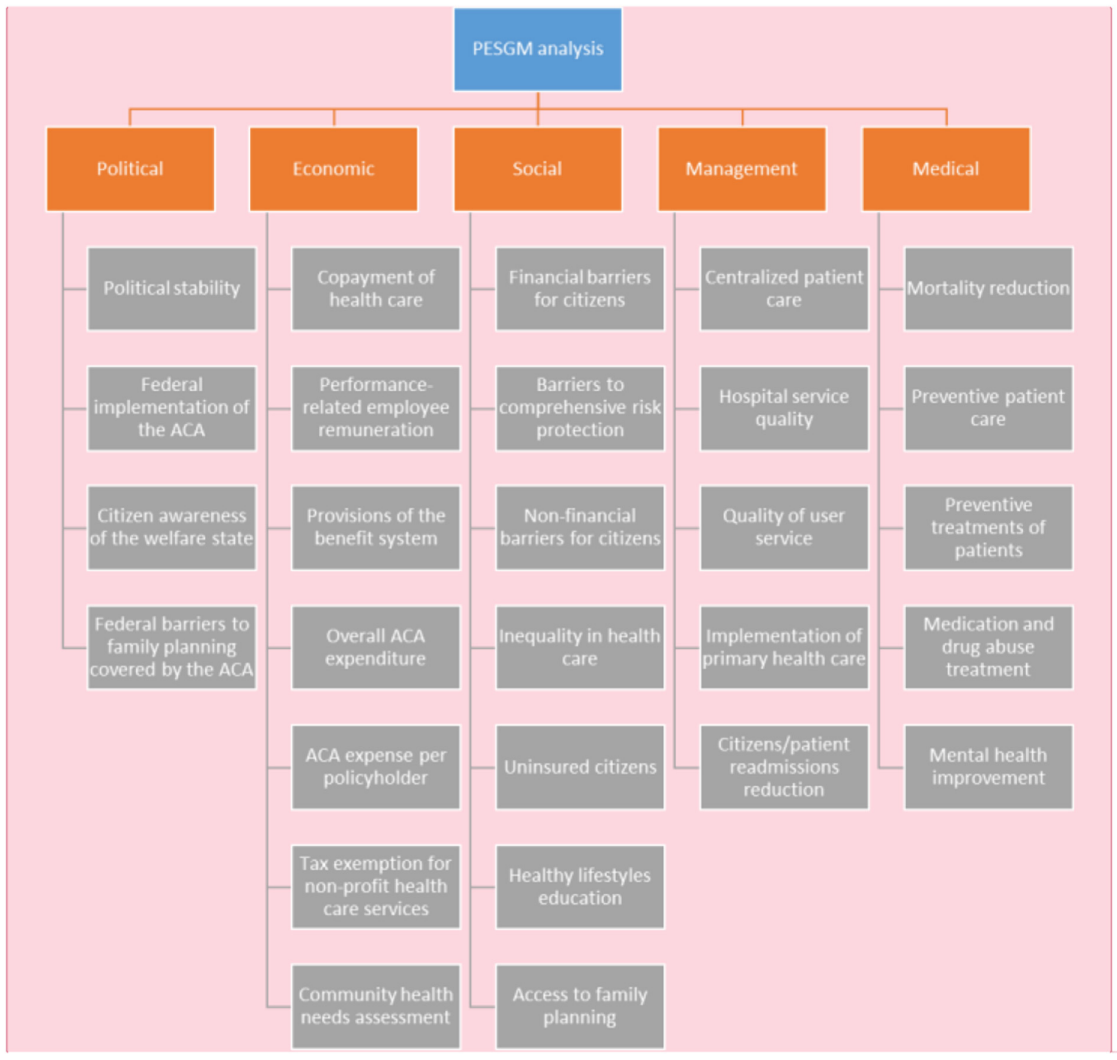
PESMM analysis of the ACA.

The results of the analysis will be presented in this section, in particular in terms of the topics that have been identified. As mentioned above, we have focused on five major topic areas of the ACA: Political, Economic, Social, Management, and Medical. Therefore, we will highlight the most significant factors of this PESMM analysis based on the bibliometric analysis carried out for each of the areas involved. In this case, no distinction will be made between periods, although we will try to use, as far as possible, the findings of authors from the last period in order to make them more up-to-date. This PESMM analysis is shown schematically in Figure 9.

The most important factors in each area are explained below:

- **Political**. Political stability is a critical factor in the ultimate success of ACA implementation. As mentioned in Section Topic analysis, the change of administration has caused changes in the course of the law and the uncertainty surrounding it, although it now seems to be entrenched thanks to Biden's rise to power. Therefore, it is evident that there is a need for political consensus between Republicans and Democrats on the fundamental aspects of the ACA to avoid jeopardizing it in the future. One of the things the Democrats should do to achieve this is to stop the media hoarding of Obamacare, which even goes by the name of the president who initiated it (Obamacare). Republicans, in turn, must understand that in the twenty-first century quality universal health care is a social need, especially in the wake of the Covid-19 health crisis. It is important to take into account the federal implementation of the law, especially in the development of Medicaid (which insures the most vulnerable classes). In 2017, nearly 20 states opted not to apply it (122), which is a significant problem for the ACA. There have also been federal barriers to implementing the law because although the law covers women's needs (including contraceptive measures and abortion), the states' own federal laws in many cases impede its application. In the last period analyzed, an issue related to public opinion arose, and of course, public awareness of the culture of free, quality universal health care is a fundamental factor for ensuring its continued existence in the future.
- **Economic**. The ACA has meant that the healthcare co-payment and co-insurance formulas have become popular and coexist on a regular basis. The ACA has incorporated formulas for a number of provisions of value-based or pay-for-performance benefit schemes. According to some authors, it is not clear that this promotes equity of care and there is a risk that resources could be diverted from hospitals and physicians serving disadvantaged populations (122). Non-profit hospitals in the USA must demonstrate the benefits they provide to the community in order to be granted tax exemption. This assessment is therefore very important (123). Public administrations encourage investment in health care that benefits the population, and there is also a demand for public-private partnerships (124). However, in addition to overall health spending, it is also important to consider spending per citizen.
- **Social**. In spite of the ACA, there are financial barriers to health care. Hefty costs in the form of co-payments, co-insurance, etc. hinder access to health care and often lead to financial hardship for citizens or even financial ruin (122). Barriers also exist in the disease coverage itself, with many citizens being underinsured in this regard (14). Although the ACA already reduced the number of uninsured citizens in 2017 (122), the most economically disadvantaged groups, which often coincide with African Americans, Hispanics, immigrants, undocumented migrants, etc. are often very vulnerable in this regard. Another important aspect is educating the population about a healthy lifestyle in areas such as nutrition, sports, etc. The law has improved access to contraception by making coverage mandatory for insured women (122), although as discussed above, there are federal barriers to such services.
- **Management**. The law has encouraged proactive health management through primary health care, centralized patient care and collaboration between health organizations. This should undoubtedly have an impact on the quality of these services and should improve management by avoiding readmissions and general inefficiencies.
- **Medical**. The reduction of mortality, pain treatment, preventive health care (promoting gynecological and urological check-ups, mammograms, etc.), treatment of drug and medication abuse, improvements in mental health, etc. are the objectives of the implementation of the ACA, and although there is still a long way to go, there are already a number of studies that point to improvements in these areas.

## Conclusion and future work

The reform brought about by the ACA is the most important reform that has been undertaken in the USA in recent times, with great repercussions at a political-legal, economic, social, management (or administrative), and medical (or health) level. In this paper we have tried to consider the impact of this law in all these areas from an objective point of view, as far as possible, thanks to the bibliometric methodology used. It has been noted that the law requires political consensus to be implemented in a definitive and global manner throughout the United States. A balance is yet to be struck between the coexistence of private and public health care to ensure broad social coverage without economic or other types of barriers. At a management level, we have also observed that considerable room for improvement exists in factors such as centralized patient management, which also undoubtedly has repercussions on efficiency and, therefore, economic factors. Several medical studies have shown the positive impact of ACA on various treatments and the prevention of diseases. From a more global point of view, it could be said that the ACA is causing a change of mentality in the USA, especially after COVID-19, which has highlighted the need for free and universal health care for the entire population.

In a more purely bibliometric aspect, the main contributors to ACA research have been identified in terms of authors, organizations, journals and countries. In addition, we have noted a downward trend in the scientific contribution to this law.

It should not be forgotten that since 2020 the world has been experiencing a health crisis caused by COVID-19, which has affected the very implementation and vision of the law. We believe that it is still too early to analyze the impact this may have had; therefore, this work could be updated in the near future. In this regard, the methodology used in this work is regarded positively, since it reflects several of many authors' partial assessments from a broader and supposedly objective point of view.

## Data availability statement

Publicly available datasets were analyzed in this study. This data can be found at: Scopus Google Scholar Web of Science.

## Author contributions

AC-A, JG, and RC contributed to conception and design of the study. RC organized the study. All authors contributed to manuscript revision, read, and approved the submitted version.

## Conflict of interest

The authors declare that the research was conducted in the absence of any commercial or financial relationships that could be construed as a potential conflict of interest.

## Publisher's note

All claims expressed in this article are solely those of the authors and do not necessarily represent those of their affiliated organizations, or those of the publisher, the editors and the reviewers. Any product that may be evaluated in this article, or claim that may be made by its manufacturer, is not guaranteed or endorsed by the publisher.
