# Supplementary material for: Obamacare: A bibliometric perspective
Source: Front Public Health. 2022 Aug 12;10:979064. doi: 10.3389/fpubh.2022.979064 (PMC9416003; doi:10.3389/fpubh.2022.979064)
Supplement: Supplementary file 1 [file Image_1.pdf]

## Appendix

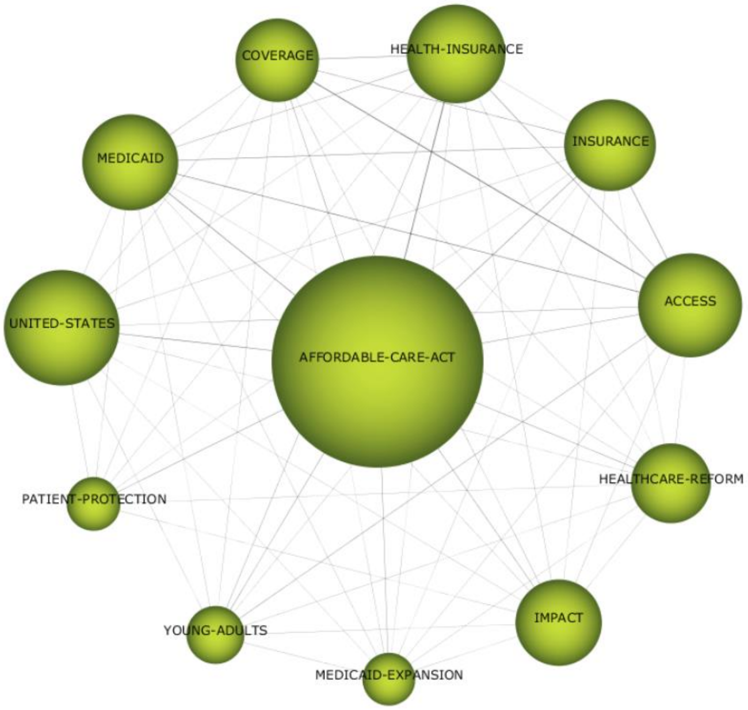


**Figure 10.** The AFFORDABLE-CARE-ACT thematic network.

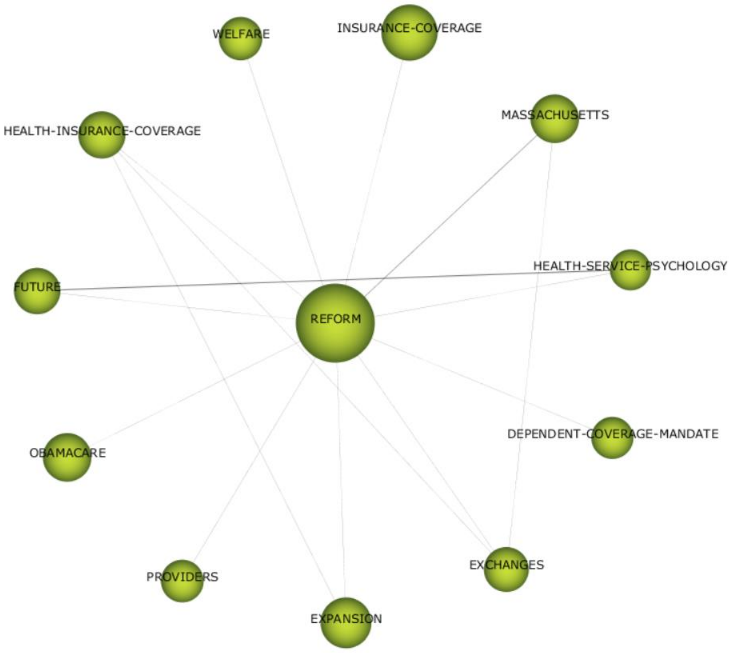


**Figure 11.** The REFORM thematic network.

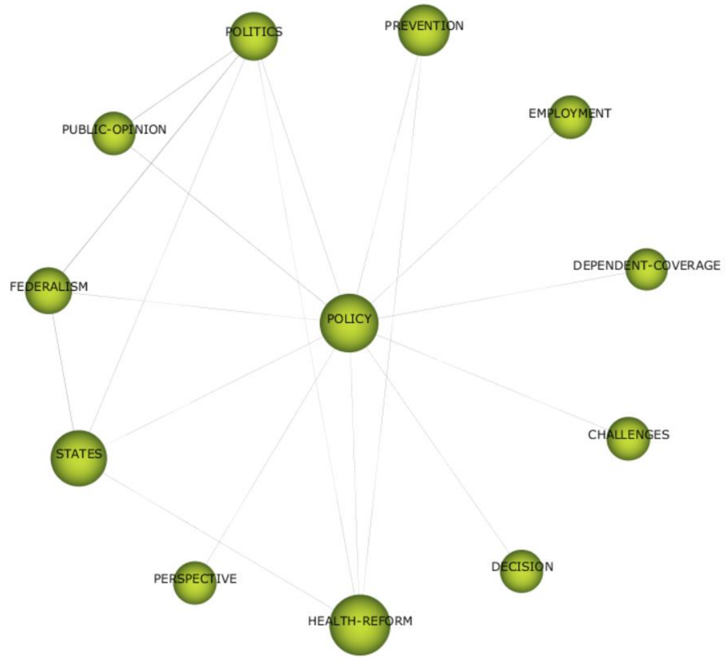


**Figure 12.** The  POLICY thematic network.

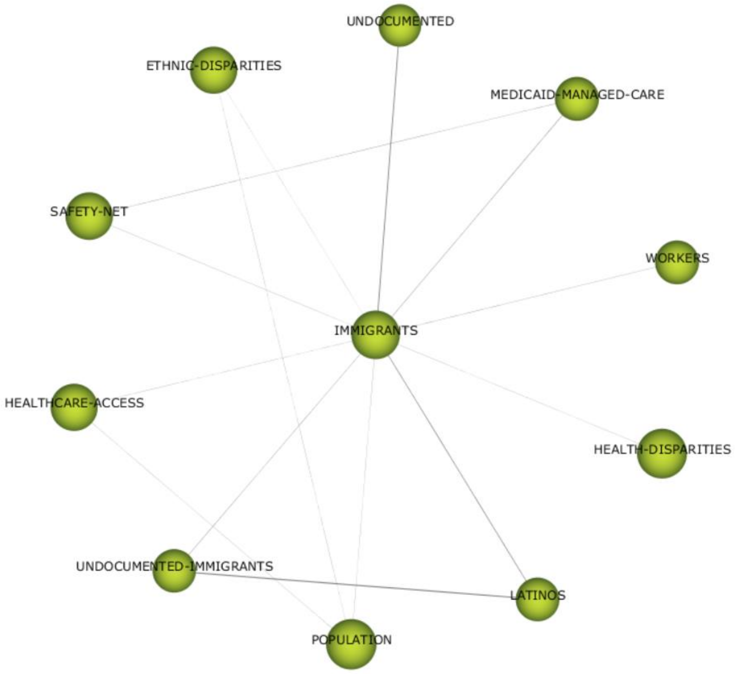


**Figure 13.** The IMMIGRANTS thematic network.

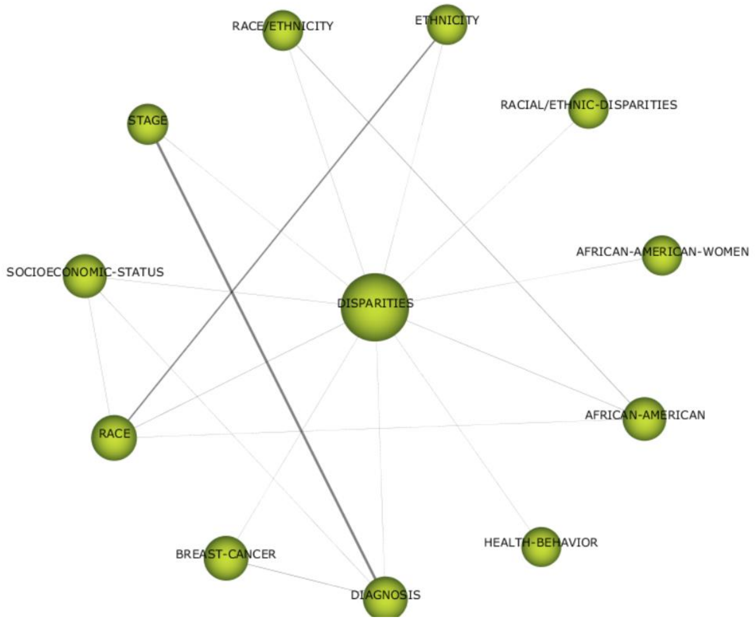


**Figure 14.** The DISPARITIES thematic network.

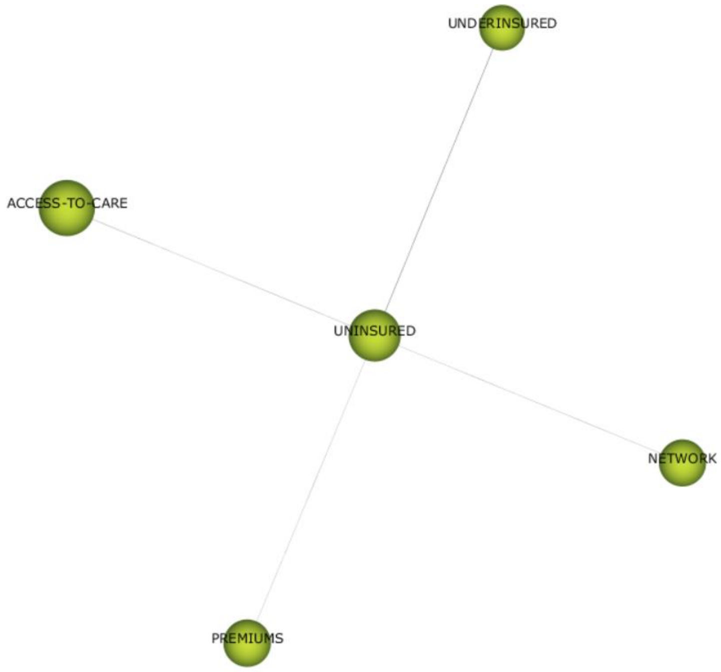


**Figure 15.** The UNINSURED thematic network.

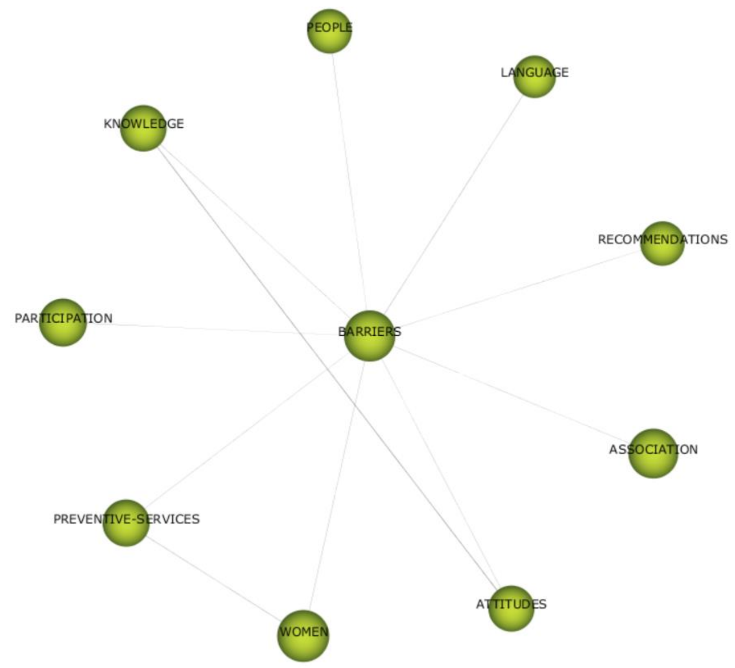


**Figure 1.** The BARRIERS thematic network.

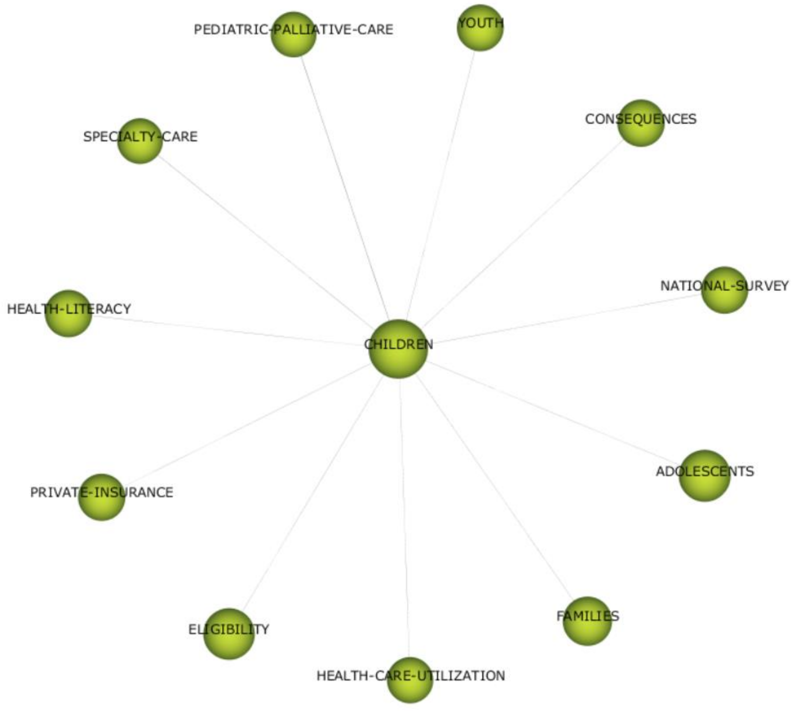


**Figure 2.** The CHILDREN thematic network.

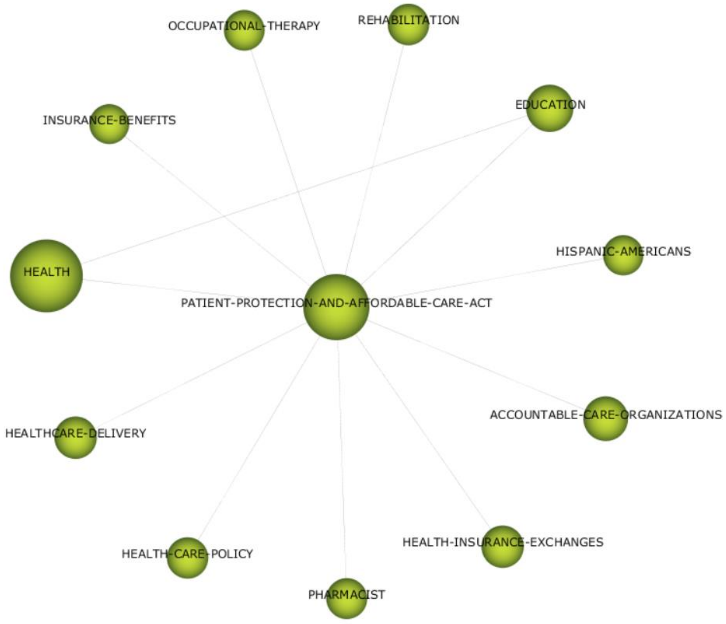


**Figure 3.** The PATIENT-PROTECTION-AND-AFFORDABLE-CARE-ACT thematic network.

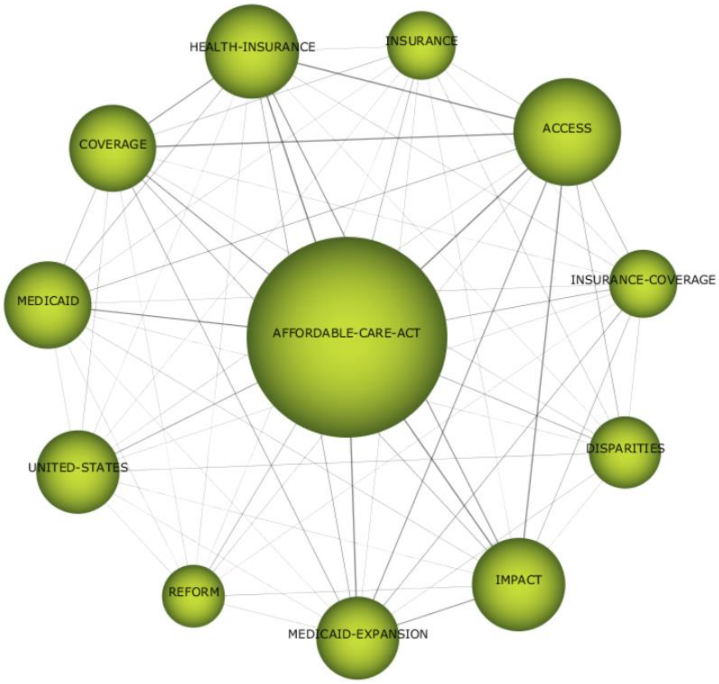


**Figure 19.** The AFFORDABLE-CARE-ACT thematic network.

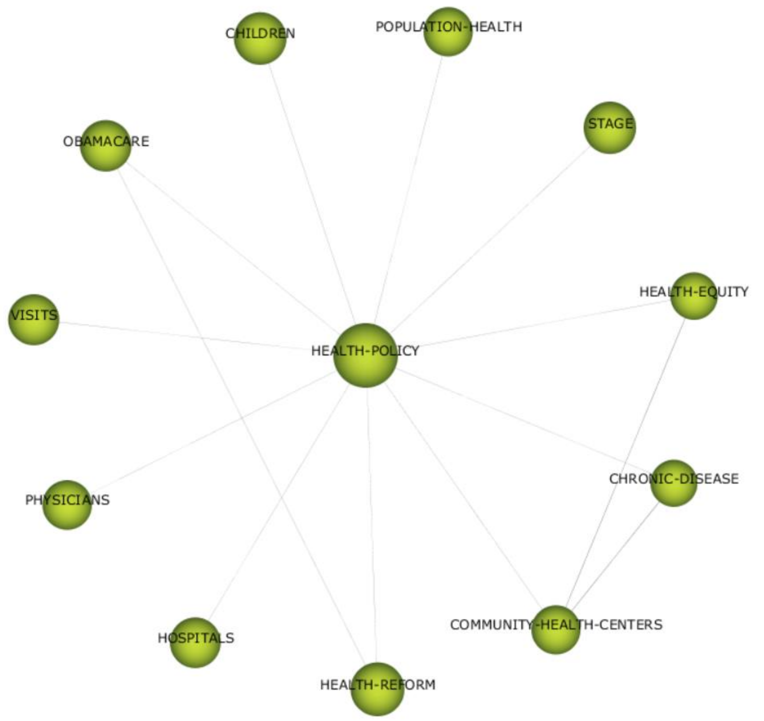


**Figure 20.** The HEALTH-POLICY thematic network from the "2018-2021" period.

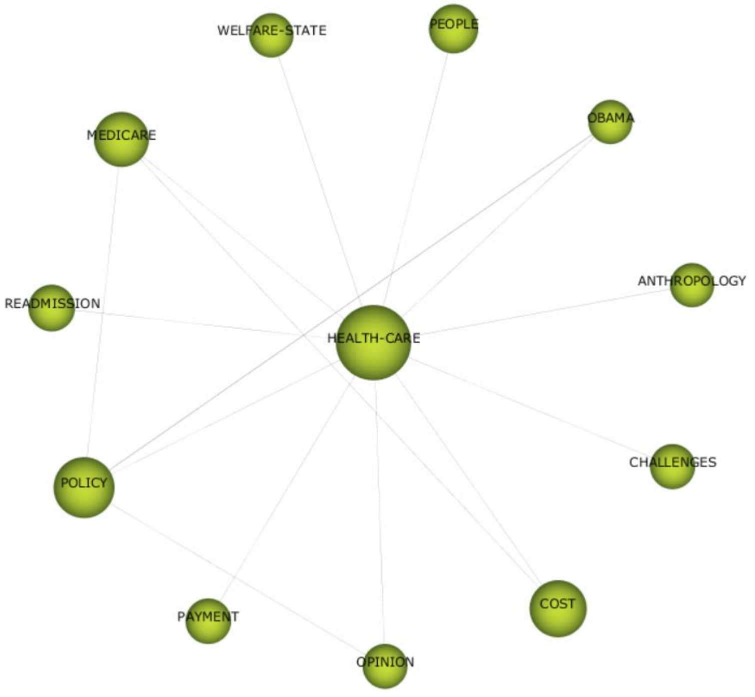


**Figure 21.** The HEALTH-CARE thematic network.

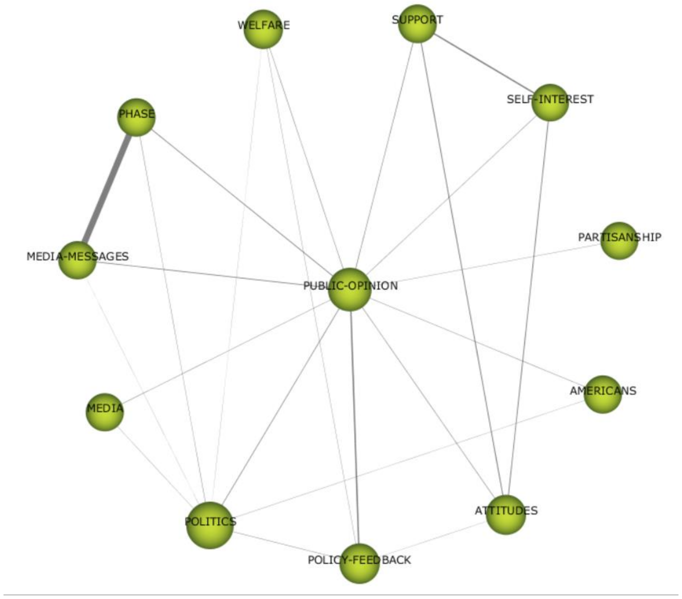


**Figure 22.** The PUBLIC-OPINION thematic network.

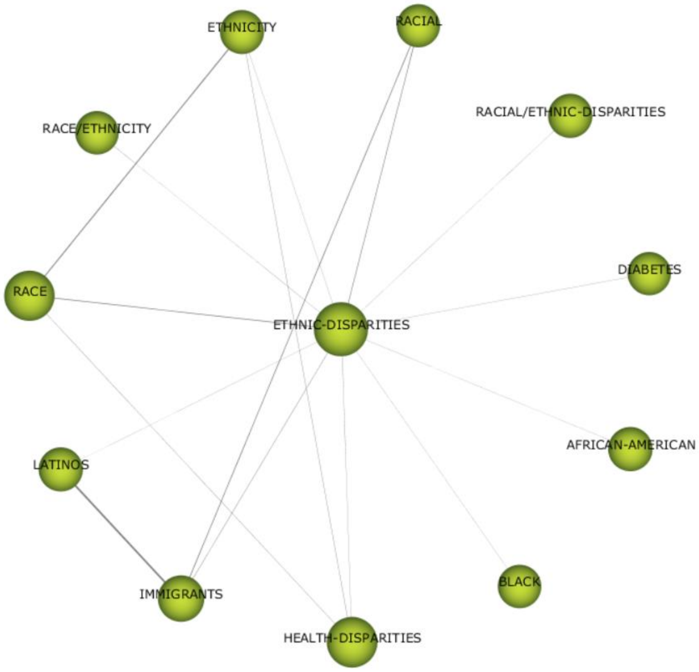


**Figure 23.** The ETHNIC-DISPARITIES thematic network.

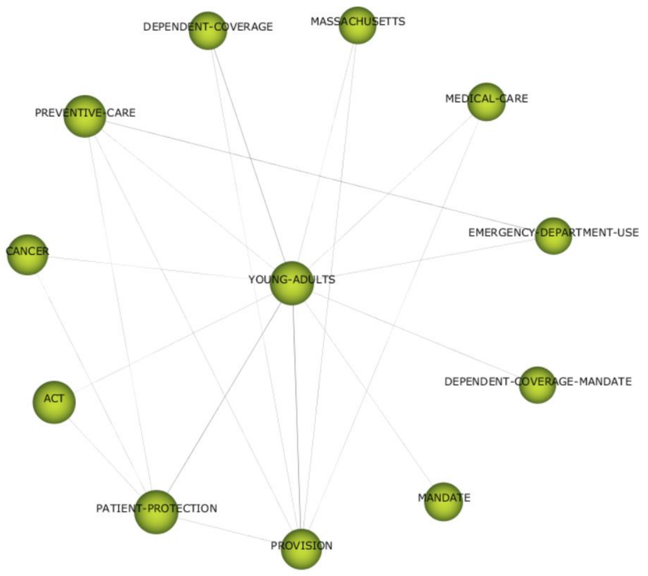


**Figure 24.** The YOUNG-ADULTS thematic network.

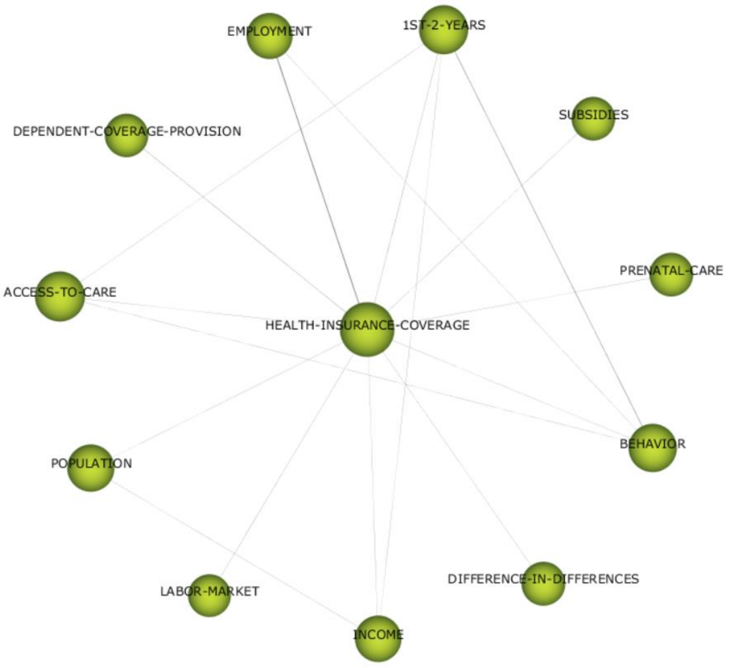


**Figure 25.** The HEALTH-INSURANCE-COVERAGE thematic network.

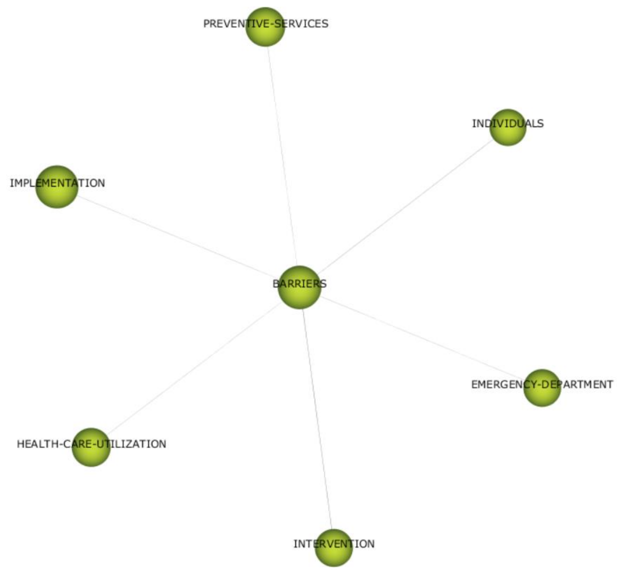


**Figure 26.** The BARRIERS thematic network.

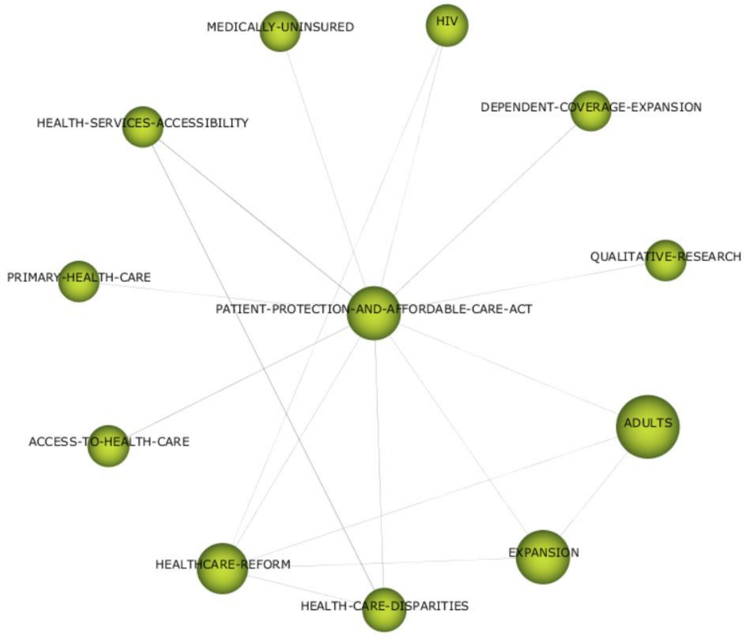


**Figure 4.** The PATIENT-PROTECTION-AND-AFFORDABLE-CARE-ACT thematic network.

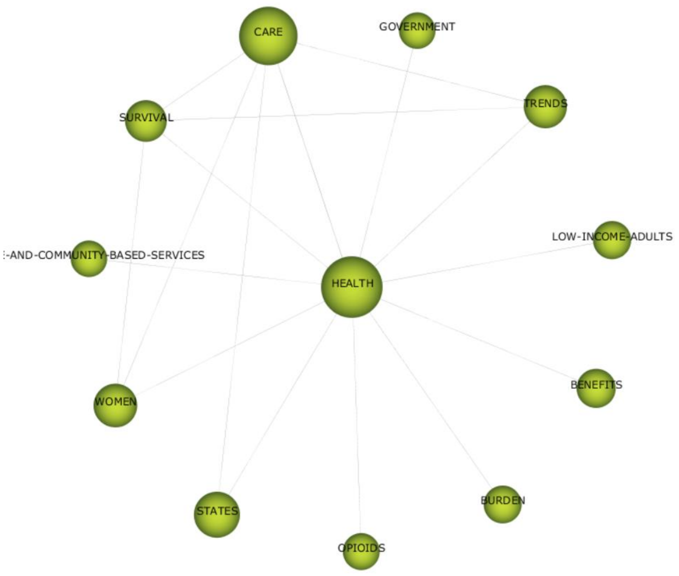


**Figure 28** The HEALTH thematic network.

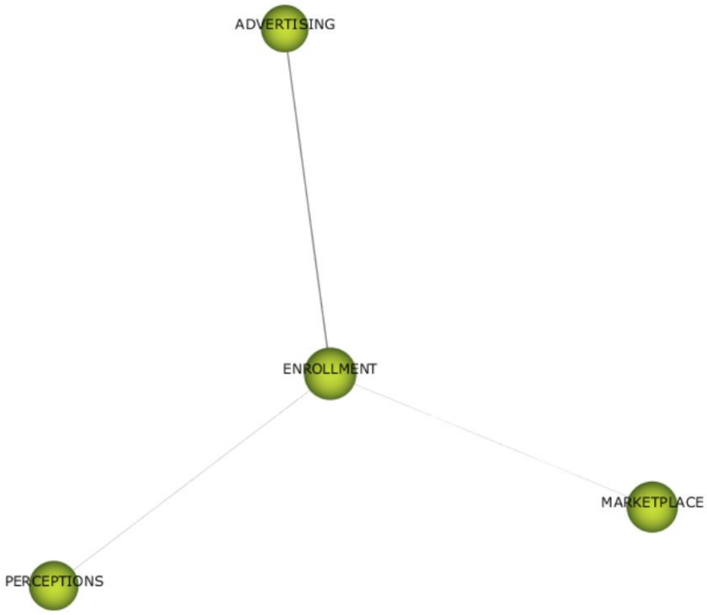


**Figure 29.** The ENROLLMENT thematic network.

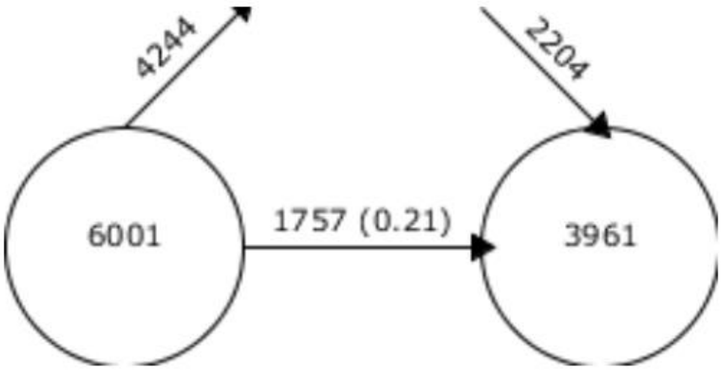


**Figure 30.** Overlap diagram from the period “Until 2017” to the period “2018-2021”.

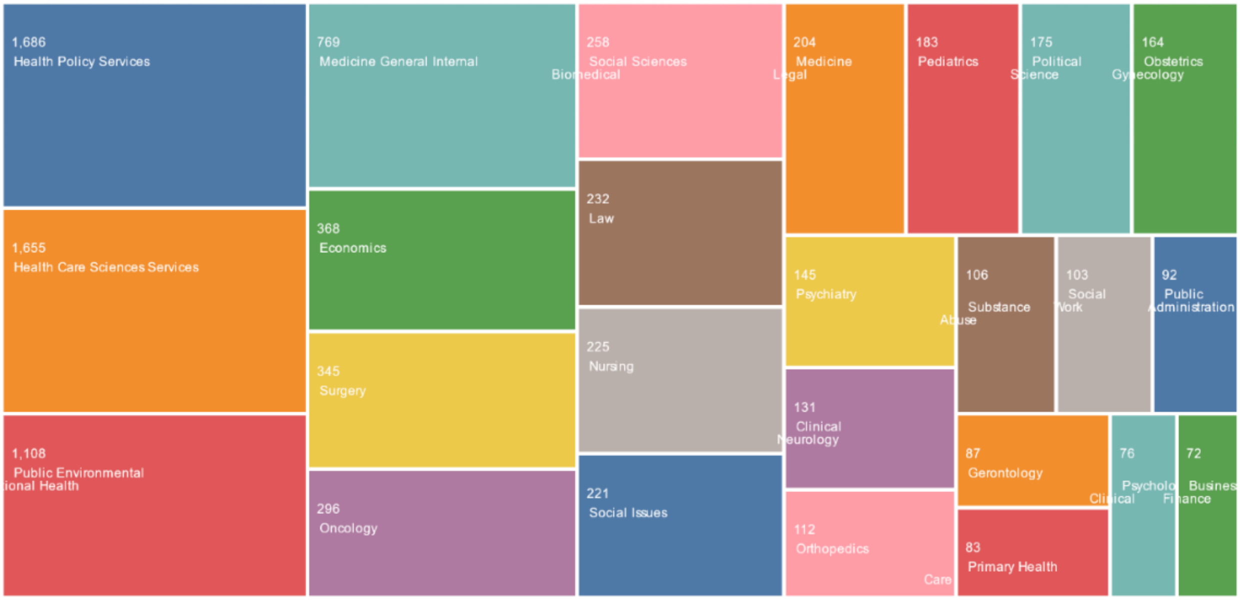


**Figure 31.** Areas of publications on the analyzed topic.

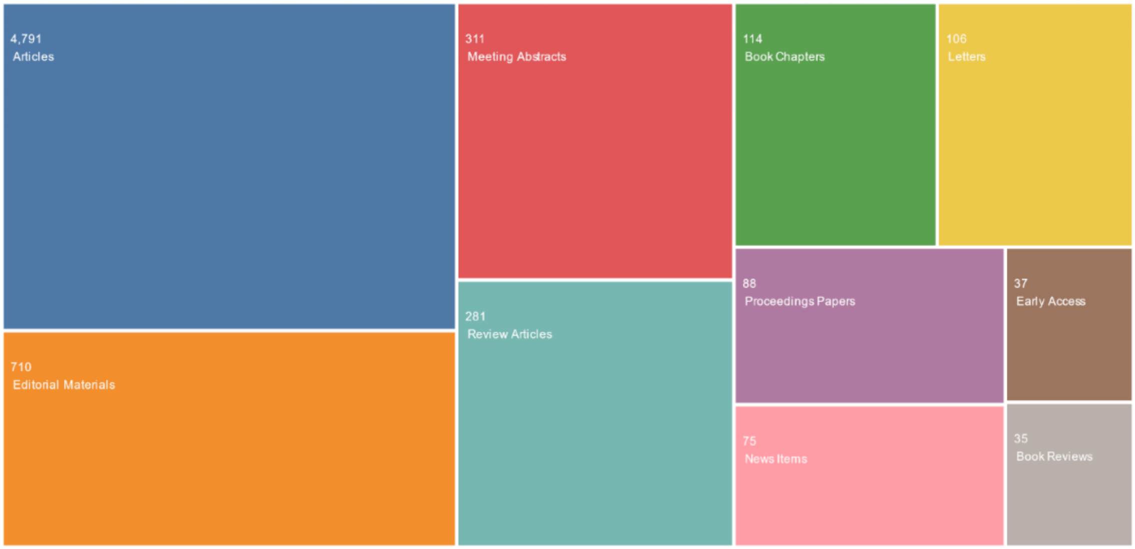


**Figure 32.** Types of publications on the analyzed topic.

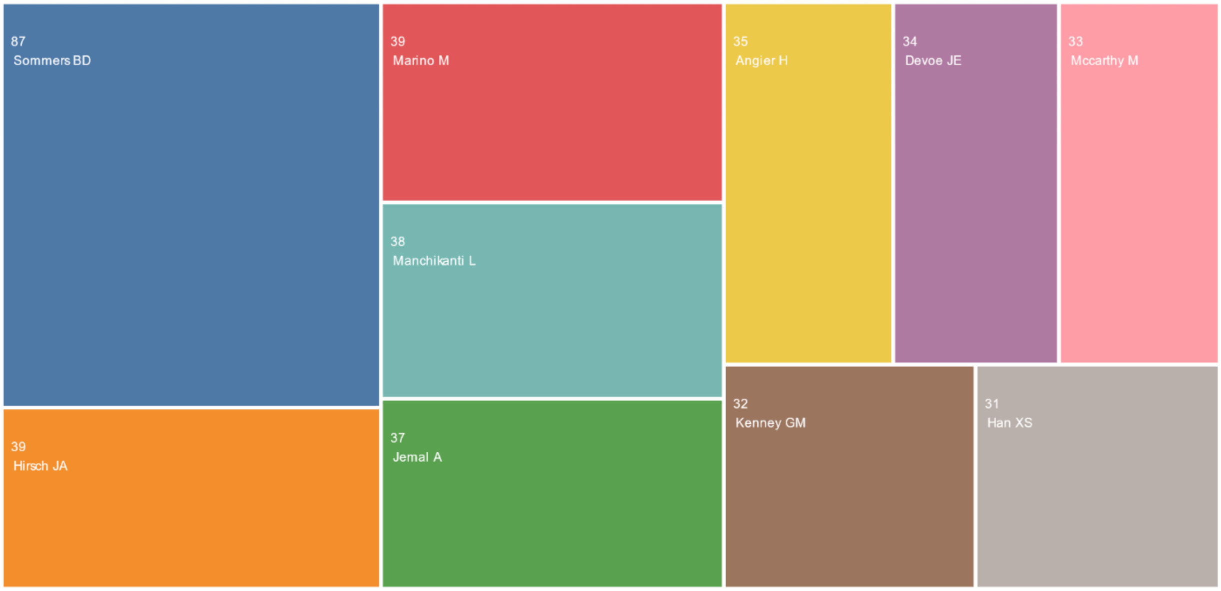


**Figure 33.** Authors with the highest number of publications on the analyzed topic.

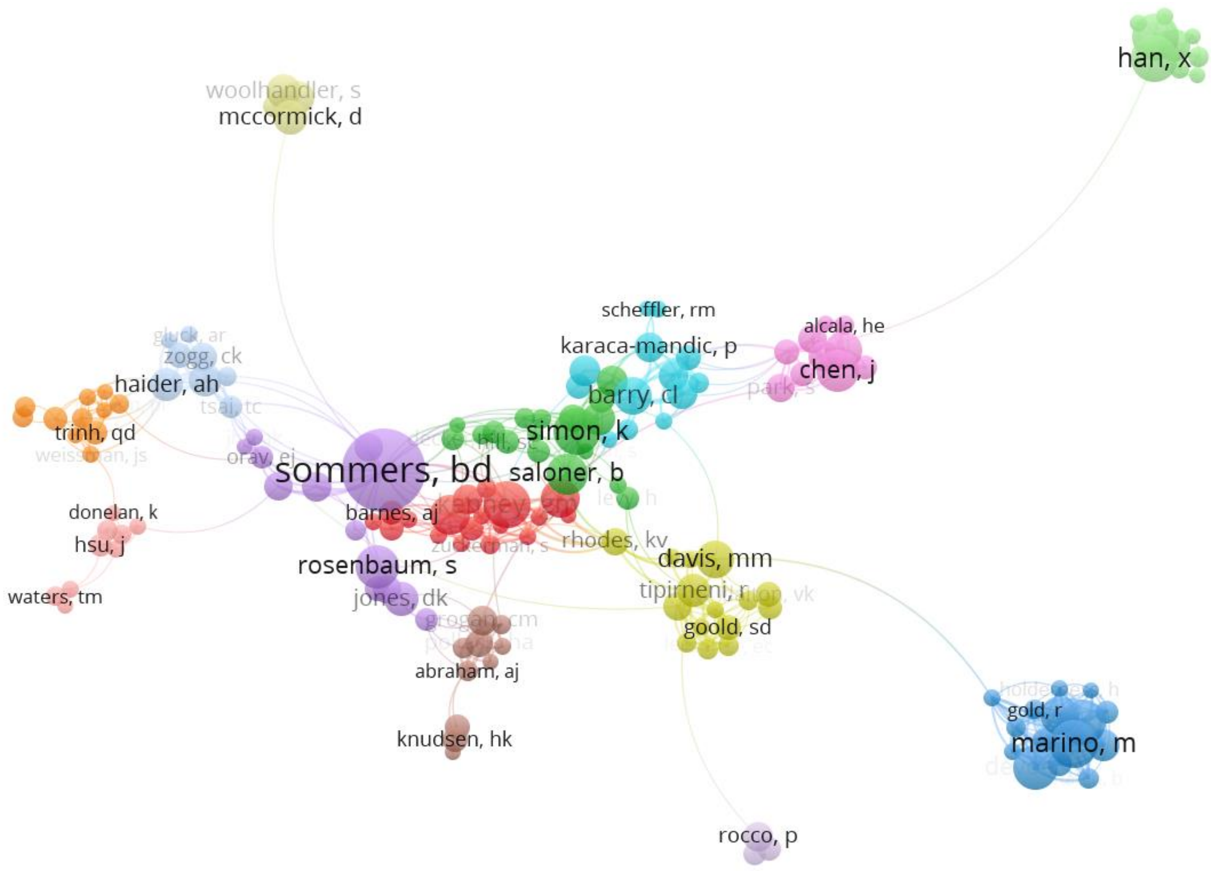


**Figure 34.** Network of co-authorships of the most important authors of the analyzed topic.

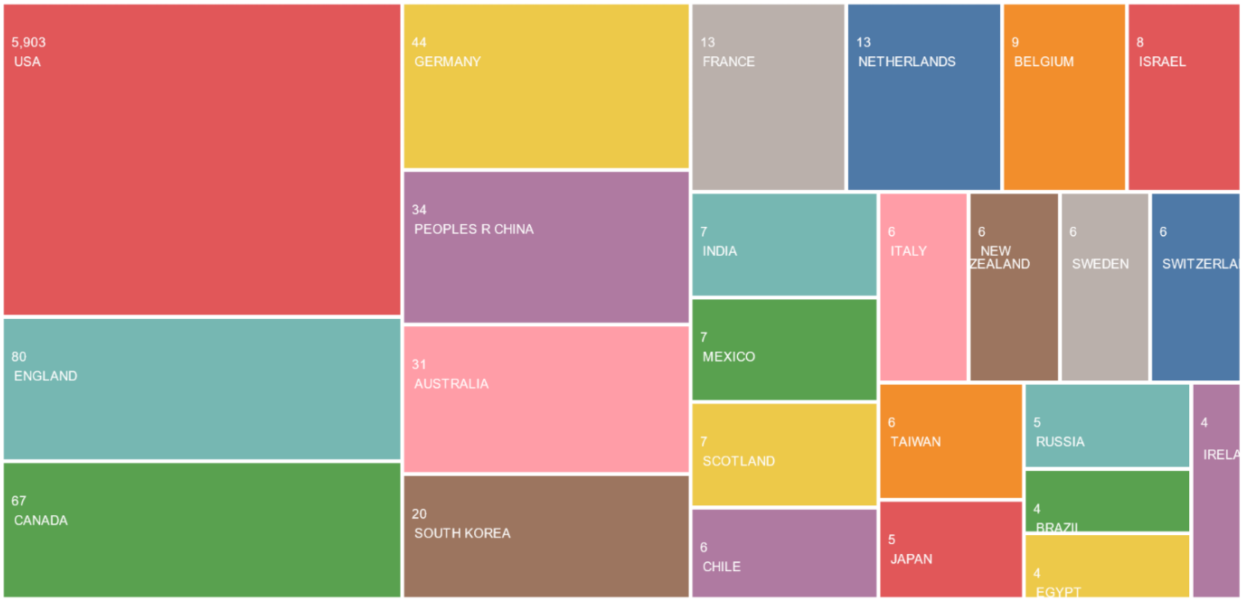


**Figure 5.** Countries with the highest number of publications on the analyzed topic.

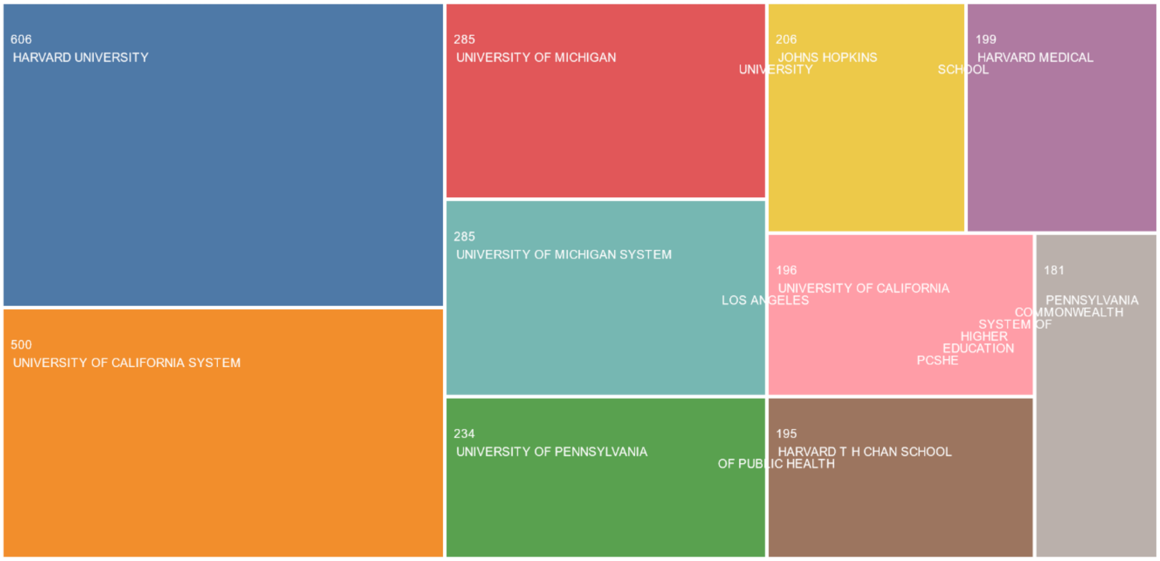


**Figure 36.** Organizations with the highest number of publications on the analyzed topic.

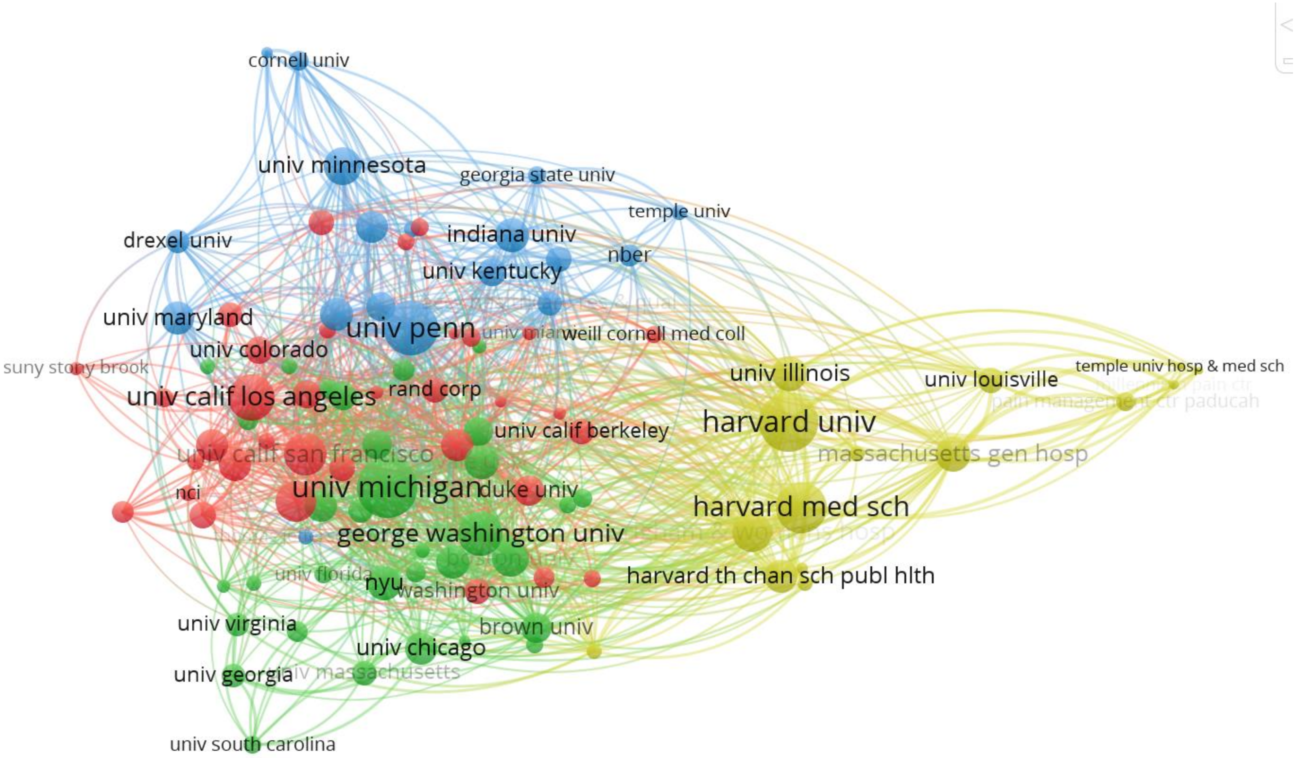


**Figure 37.** Organization analysis by author.
